# Current progress in innovative engineered antibodies

**DOI:** 10.1007/s13238-017-0457-8

**Published:** 2017-08-18

**Authors:** William R. Strohl

**Affiliations:** BiStro Biotech Consulting, Bridgewater, NJ 08807 USA

**Keywords:** antibody clinical candidates, engineered antibodies, chimeric antigen receptors

## Abstract

As of May 1, 2017, 74 antibody-based molecules have been approved by a regulatory authority in a major market. Additionally, there are 70 and 575 antibody-based molecules in phase III and phase I/II clinical trials, respectively. These total 719 antibody-based clinical stage molecules include 493 naked IgGs, 87 antibody-drug conjugates, 61 bispecific antibodies, 37 total Fc fusion proteins, 17 radioimmunoglobulins, 13 antibody fragments, and 11 immunocytokines. New uses for these antibodies are being discovered each year. For oncology, many of the exciting new approaches involve antibody modulation of T-cells. There are over 80 antibodies in clinical trials targeting T cell checkpoints, 26 T-cell-redirected bispecific antibodies, and 145 chimeric antigen receptor (CAR) cell-based candidates (all currently in phase I or II clinical trials), totaling more than 250 T cell interacting clinical stage antibody-based candidates. Finally, significant progress has been made recently on routes of delivery, including delivery of proteins across the blood-brain barrier, oral delivery to the gut, delivery to the cellular cytosol, and gene- and viral-based delivery of antibodies. Thus, there are currently at least 864 antibody-based clinical stage molecules or cells, with incredible diversity in how they are constructed and what activities they impart. These are followed by a next wave of novel molecules, approaches, and new methods and routes of delivery, demonstrating that the field of antibody-based biologics is very innovative and diverse in its approaches to fulfill their promise to treat unmet medical needs.

## INTRODUCTION

This year, 2017, marks the 20th anniversary of the approval by the United States Food and Drug Administration (US FDA) of Rituxan® (rituximab) and Zenapax® (daclizumab), for treatment of B cell malignancies and for use to suppress organ rejection in renal transplants, respectively (Table 1). While two antibodies had previously been approved by the FDA (Table 1), the approval of Rituxan® and Zenapax® in 1997 was a watershed moment in the history of monoclonal antibody (mAb) therapeutics. The reasons are very different for each molecule. Rituxan® has become both a huge medical and commercial success, with indications in B cell malignancies as well as in the treatment of rheumatoid arthritis (RA) (Storz, 2014). Rituxan® is currently the fourth best-selling innovative drug of any kind with 2016 worldwide sales of $8,354 MM (Table 2), about 85% of those sales coming in cancer indications and the other 15% from sales for treatment of RA (La Merie Publishing, 2017). Including Rituxan®, seven of the top ten selling innovative drugs in the world in 2016 were proteins, six of which were antibody- related molecules (Table 2). Zenapax®, on the other hand, was the first humanized antibody to be FDA approved but it never achieved significant commercial success and was eventually withdrawn from the market in 2009. Daclizumab, however, has been approved recently under the tradename Zinbryta® for treatment of relapsing forms of multiple sclerosis (MS).

**Table 1:** Innovative monoclonal antibodies and fusion proteins approved for marketing in European Union, United States, or Japan*

US trade name (Generic name)	Company	Approval date (US)**	Molecular target***	Major indication (s)	Protein format	Source of variable sequences**
1. Orthoclone OKT3® (Muromonab-CD3)	Ortho Biotech (J&J)	06/19/86; withdrawn 2011	CD3E (CD3ɛ)	OTR	Murine IgG2a	Mouse hybridoma
2. ReoPro® (Abciximab)	Centocor (now J&J)/Lilly	12/22/94	ITGA2B/ITGB3 (gPIIb/IIIa)	CVD	Chimeric FAb fragment	Mouse hybridoma
3. Rituxan® (Rituximab)	Biogen/Idec/Genentech	11/26/97	MS4A1 (CD20)	NHL, RA	Chimeric IgG1	Mouse hybridoma
4. Zenapax® (Daclizumab)	Biogen/Abbott (PDL/Roche)	12/10/97; withdrawn 2009	IL2RA (IL-2Rα; CD25)	OTR	Humanized IgG1	Mouse hybridoma
5. Remicade® (Infliximab)	Centocor (now J&J)	8/24/98	TNF (TNF-α)	CRD, RA	Chimeric IgG1	Mouse hybridoma
6. Synagis® (Palivizumab)	MedImmune	06/19/98	RSV F-protein	RSV infection	Humanized IgG1	Mouse hybridoma
7. Herceptin® (Trastuzumab)	Genentech	09/25/98	ERBB2 (HER2)	Breast cancer	Humanized IgG1	Mouse hybridoma
8. Enbrel® (Etanercept)	Immunex (now Amgen)	11/02/98	TNF (TNF-α)	RA	P75-TNFR-Fc fusion	Fc fusion
9. Simulect® (Basiliximab)	Novartis	12/05/98	IL2RA (IL-2Rα; CD25)	OTR	Chimeric IgG1	Mouse hybridoma
10. Mylotarg® (Gemtuzumab ozogamicin)	Wyeth (now Pfizer)	05/17/00; withdrawn 2010	CD33	Leukemia	Humanized IgG4-ADC****	Mouse hybridoma
11. Campath -1H® (Alemtuzumab)	Genzyme	05/07/01; withdrawn 2012	CD52	Leukemia	Humanized IgG1	Rat hybridoma
12. Zevalin® (Ibritumomab tiuxetan)	Biogen/Idec	02/19/ 2002	MS4A1 (CD20)	NHL	Murine IgG1 radio-conjugate (Y-90,In-111)	Mouse hybridoma
13. Humira® (Adalimumab)	CAT, Abbott	12/31/02	TNF (TNF-α)	RA, CRD	Human IgG1	Human antibody phage library
14. Amevive® (Alefacept)	Biogen	01/30/03	CD2	Psoriasis	CD58 (LFA-3)-Fc fusion	Fc fusion
15. Xolair® (Omalizumab)	Genentech	06/20/03	IGES (IgE)	Asthma	Humanized IgG1	Mouse hybridoma
16. Bexxar® (Tositumomab-I131)	Corixa	06/27/03; withdrawn 2014	MS4A1 (CD20)	NHL	Murine IgG2a radio-conjugate (I-131)	Mouse hybridoma
17. Raptiva® (Efalizumab)	Genentech	10/27/03; withdrawn 2009	ITGAL (CD11A)	Psoriasis	Humanized IgG1	Mouse hybridoma
18. Erbitux® (Cetuximab)	ImClone/BMS	02/12/04	EGFR	CRC	Chimeric IgG1	Mouse hybridoma
19. Avastin® (Bevacizumab)	Genentech	02/26/04	VEGFA	CRC	Humanized IgG1	Mouse hybridoma
20. Tysabri® (Natalizumab)	Biogen/Elan	11/23/04	ITGA4 (α4 integrin)	MS	Humanized IgG4	Hybridoma
21. Orencia® (Abatacept)	BMS	12/23/05	CD80/CD86	RA	CTLA4-Fc fusion	Fc fusion
22. Lucentis® (Ranibizumab)	Genentech Novartis	06/30/06	VEGFA	Wet AMD	Humanized Fab fragment	Hybridoma
23. Vectibix® (Panitumumab)	Amgen	09/27/06	EGFR	Colorectal cancer	Human IgG2	TG Xenomouse
24. Soliris® (Eculizumab)	Alexion Pharma	03/16/07	C5	PNH	Humanized hybrid engineered IgG2/4	Mouse hybridoma
25. Arcalyst® (Rilonacept)	Regeneron	02/27/08	IL1A (IL-1α), IL1B (IL-1β), IL1RN (IL-1RA)	CAPS, MWS	IL-1R & IL-1AP-in-line Fc fusion	Fc fusion
26. Nplate® (Romiplostim)	Amgen	08/22/08	MPL (TPO-R)	Thrombo-cytopenia	Fc-peptide fusion (“peptibody”)	Peptide phage library
27. Simponi® (Golimumab)	Centocor/J&J	04/23/09	TNF (TNF-α)	RA	Human IgG1	HuMAb TG mouse
28. Stelara® (Ustikinumab)	Centocor/J&J	09/25/09	IL12B (p40 subunit of IL-12 and IL-23)	Psoriasis	Human IgG1	HuMAb TG mouse
29. Removab® (Catumaxomab)	Fresenius/Trion	EU only 4/23/09; withdrawn 2017	EPCAM, CD3E	Malignant ascites	Rat IgG2b-mouse IgG2a hybrid bispecific IgG	Mouse and rat hybridomas
30. Cimzia® (Certolizumab pegol)	UCB/Schwartz	05/14/09	TNF (TNF-α)	RA	PEGylated humanized FAb fragment	Mouse hybridoma
31. Ilaris® (Canakinumab)	Novartis	06/19/09	IL1B (IL-1β)	CAPS	Human IgG1	HuMAb TG mouse
32. Arzerra® (Ofatumumab)	GenMab/Novartis#	10/26/09	MS4A1 (CD20)	CLL	Human IgG1	HuMAb TG mouse
30. Actemra® (Tocilizumab)	Roche/Chugai/Genentech	01/09/10	IL6R (CD126)	MCD; RA	Humanized IgG1	Hybridoma
31 Prolia®/Xgeva® (Denosumab)	Amgen/GSK	06/01/10	TNFSF11 (RANK-ligand)	Osteoporosis, Bone cancer	Human IgG2	TG Xenomouse
35. Benlysta® (Belimumab)	GSK/HGS	03/09/11	TNFSF13B (soluble BLyS)	SLE	Human IgG 1	Human antibody phage library
36. Yervoy® (Ipilimumab)	Medarex/BMS	03/25/11	CTLA4	Melanoma	Human IgG1	HuMAb TG mouse
37. Nuloji® (Belatacept)	BMS	06/16/11	CD80/CD86	OTR	CTLA-4 Fc fusion	Fc fusion
38. ADCETRIS® (Brentuximab vedotin)	Seattle Genetics/Takeda/Millenium	08/19/11	TNFRSF8 (CD30)	Hodgkin’s lymphoma	Chimeric IgG1 ADC****	Mouse hybridoma
39a. EYLEA® (aflibercept)	Bayer-Schering/Regeneron	11/18/11	VEGFA	Wet AMD	VEGF-R-Fc fusion	Fc fusion
40. POTELIGEO® (Mogamulizumab)	Kyowa Hakko Kirin	Japan only 03/30/12	CCR4	ATL	Humanized IgG1-Afucosylated glycan	Mouse hybridoma
41. Perjeta® (Pertuzumab)	Genentech	06/08/12	ERBB2 (HER2)	Breast cancer	Humanized IgG1	Mouse hybridoma
(39b). ZALTRAP® (ziv-aflibercept)	Sanofi/Regeneron	08/03/12	VEGFA	MCRC	VEGFR-Fc fusion protein Trap	Fc fusion
42. Abthrax® (Raxibacumab)	GSK; Human Genome Sciences	12/14/12	*Bacillus anthracis* PA toxin	Anthrax biodefense	Human IgG1	Human antibody phage library
43. Kadcyla® (trastuzumab emtansine)	Roche/Genentech	02/23/13	ERBB2 (HER2)	Breast cancer	Humanized IgG ADC****	Mouse hybridoma
44. Gazyva® (obinutuzumab)	Roche/Genentech/Biogen	11/01/13	MS4A1 (CD20)	CLL	Humanized IgG1-low fucose	Mouse hybridoma
45. Alprolix**®** (Eftrenonacog alfa)	Biogen-IDEC/Biovitrum	03/28/14	Factor substitute	Hemophilia B	Monomeric Factor IX Fc usion protein	Fc fusion
46. Cyramza® (Ramucirumab)	Lilly/Dyax	04/22/14	KDR (VEGFR-2)	Gastric cancer	Human IgG1	Human antibody phage library
47. Sylvant® (Siltuximab)	Janssen R&D/J&J	04/23/14	IL6	MCD	Chimeric IgG1	Mouse hybridoma
48. Entyvio® (vedolizumab)	Takeda/Millenium	05/20/14	ITGA4/ITGB7 (α4β7 integrin)	CRD	Humanized IgG1	Mouse hybridoma
49. Eloctate® (Efmoroctocog alfa)	Biogen Idec/SOBI	06/06/14	Factor substitute	Hemophilia A	Monomeric Fc domain-deleted F-VIII fusion	Fc fusion
50. Keytruda® (pembrolizumab)	Merck	09/04/14	PDCD1 (PD-1)	Melanoma	Humanized IgG4	Mouse hybridoma
51. Trulicity® (dulaglutide)	Eli Lilly	09/18/14	GLP1R (agonist)	Type 2 diabetes	GLP-1 – Fc fusion	Fc fusion
(11). Lemtrada® (alemtuzumab)	Genzyme (Sanofi subsidiary)	11/14/14	CD52	MS	Humanized IgG1	Rat hybridoma
52. Blincyto® (blinatumomab)	Amgen (Micromet)	12/03/14	CD19, CD3E	B-cell ALL	BiTE	Mouse hybridoma
53. Opdivo® (nivolumab)	BMS	12/22/14	PDCD1 (PD-1)	Melanoma	Human IgG4	HuMAb TG mouse
54. Cosentyx® (secukinumab)	Novartis	01/21/15	IL17A	Plaque psoriasis	Human IgG1	HuMAb TG mouse
55. Unituxin® (dinutuximab)	United Technologies/NCI	03/10/15	GD2	Neuroblastom a	Chimeric IgG1	Mouse
56. Praluent® (alirocumab)	Sanofi/Regeneron	07/24/15	PCSK9	High cholesterol	Human IgG1	VelocImmune TG mouse
57. Repatha® (evolocumab)	Amgen (Astellas in Japan)	08/27/15	PCSK9	High cholesterol	Human IgG1	TG Xenomouse
58. Praxbind® (idarucizumab)	Boerhinger Ingelheim	10/16/15	Dabigatran	Drug Reversal	Humanized Fab fragment	Mouse hybridoma
59. Strensiq® (Asfotase alfa)	Alexion (from Enobia)	10/23/15	Factor substitute	Hypophos-phatasia	TNSALP - Fc fusion-peptide	Fc fusion
60. Nucala® (Mepolizumab)	GSK	11/06/15	IL5	COPD	Humanized IgG1	Mouse hybridoma
61. Darzalex® (daratumumab)	Janssen R&D (J&J)/Genmab	11/16/15	CD38	MM	Human IgG1	HuMAb TG mouse
62. Portrazza® (necitumumab)	Lilly/ImClone/Dyax	11/24/15	EGFR	Squamous NSCLC	Human IgG1	Human antibody phage library
63. Empliciti® (elotuzumab)	BMS/ Abbvie (from PDL)	11/30/15	SLAMF7	MM	Humanized IgG	Mouse hybridoma
64. Anthim® (obiltoxaximab)	Elusys Therapeutics	03/21/16	*Bacillus anthracis* PA toxin	Anthrax-biodefense	Chimeric IgG	Mouse hybridoma
65. Taltz® (Ixekizumab)	Eli Lilly	03/22/16	IL17A	Psoriasis; PsA	Humanized IgG4	Mouse hybridoma
66. Cinqair® (Reslizumab)	Teva Ception/Cephalon	03/23/16	IL5	Eosinophilic asthma	Humanized IgG4	Rat hybridoma
67. Tecentriq® (Atezolizumab)	Roche/Genentech	05/18/16	CD274 (PD-L1, B7-H1)	Bladder cancer	Humanized IgG1	Mouse hybridoma
(4). Zinbryta® (Daclizumab)	Biogen/Abbott (PDL/Roche)	May 2016	IL2RA (IL-2Rα; CD25)	RR-MS	Humanized IgG1	Mouse hybridoma
68. Lartruvo™ (Olaratumab)	Lilly/ImClone	10/19/16	PDGFRA	Soft tissue sarcoma	Human IgG1	UltimAb TG mouse
69. Zinplava™ (Bezlotoxumab)	Medarex/MBL/Merck	10/22/16	*Clostridium difficile* B toxin	CDAD	Human IgG1	HuMAb TG mouse
70. Siliq™ (Brodalumab)	Valeant/AstraZeneca	02/15/17	IL17RA	Psoriasis	Human IgG	TG Xenomouse
71. Bavencio™ (Avelumab)	Pfizer/Merck KGaA (EMD Serono)/Dyax	3/23/17	CD274 (PD-L1, B7-H1)	Merkel cell carcinoma	Human IgG1	Human antibody phage library
72. Dupixent® (Dupilumab)	Regeneron/Sanofi	3/28/17	IL4R	Atopic dermatitis	Human IgG4 S/P	VelocImmune TG mouse
73. Ocrevus™ (Ocrelizumab)	Roche/Biogen	3/28/17	MS4A1 (CD20)	Primary, progressing MS	Humanized IgG1	Mouse hybridoma
74. Imfinzi™ (Durvalumab)	AstraZeneca (MedImmune)/Celgene	5/1/17	CD274 (PD-L1, B7- H1)	Metastatic urothelial carcinoma	Human IgG1	TG Xenomouse

Abbreviations: ADC, antibody-drug conjugate; AMD, Age-related macular degeneration; ATL, adult T-cell leukemia/lymphoma; BiTE, bispecific T cell engager; BlyS, B lymphocyte stimulator; C5, complement component C5; CAPS, Cropyrin-associated periodic syndrome; CCR4, C-C motif receptor-4; CD, cluster of differentiation; CDAD, *Clostridium difficile*-associated disease; CLL, chronic lymphocytic leukemia; COPD, chronic obstructive pulmonary disease; CRC, colorectal cancer; CRD, Crohn’s Disease; CTLA4, cytotoxic T-lymphocyte associated protein-4; CVD, cardiovascular disease; EGFR, epidermal growth factor receptor; ERBB2, erb-b2 receptor tyrosine kinase 2; F-VIII, Factor VIII; Fab, fragment, antigen-binding; Fc, fragment, crystallizable; GD2, disialoganglioside-2; GLP-1R, glucagon-like peptide-1 receptor; I-131, Iodine-131 (radioactive); HER2, human epidermal growth factor receptor-2; Ig, immunoglobulin; IL, interleukin; KDR, kinase insert domain receptor; LFA, lymphocyte- associated antigen; MCD, multicentric Castleman’s disease; MCRC, metastatic colorectal cancer; MM, multiple myeloma; MPL, myeloproliferative leukemia virus oncogene; MS, multiple sclerosis; MWS, Muckle-Wells syndrome; ND, not disclosed; NHL, non-Hodgkin lymphoma; NSCLC, non-small cell lung cancer; OTR, organ transplant rejection; PA, protective antigen; PCSK9, Proprotein convertase subtilisin/kexin type 9; PDCD1, programmed cell death 1; PDGFR, platelet-derived growth factor receptor; PD-L1, programmed cell death protein ligand-1; PEG, poly-ethylene-glycol; PNH, paroxysmal nocturnal hemoglobinuria; PsA, psoriatic arthritis; RA, rheumatoid arthritis; RANK, receptor activator of nuclear factor kappa-B; RR-MS, relapsing-remitting multiple sclerosis; RSV, respiratory syncytial virus; SC, subcutaneous; SLAMF7, signaling lymphocytic activation molecule family member 7; SLE, systemic lupus erythematosus; S/P, mutations in hinge of IgG4; TG, transgenic (humanized); TNALP, tissue-nonspecific alkaline phosphatase; TNF, tissue necrosis factor; TPO-R, thrombopoietin receptor; VEGF, vascular endothelial growth factor

* Data obtained from Prescribing Information released by the manufacturers, Company websites, AdisInsights, and BiStro Biotech Consulting database on clinical stage biologics

** US FDA approval dates unless otherwise stated

*** Names given as HUGO Gene Nomenclature Committee (HGNC) names (Gray et al., 2015) followed by commonly used names in parentheses

**** Conjugates: Mylotarg®, calicheamicin; Adcetris®, monomethyl auristatin E (MMAE); Kadcyla®, maytansanoid DM-1

# Currently not being marketed; clinical trials in MS suggest a probable relaunch in a new therapeutic area soon

**Table 2:** Top ten best-selling innovative drugs worldwide in 2016*

#	Drug (generic name)	Class	Molecular target**	Company	Primary indications (abbreviated)	2015 worldwide sales	2016 worldwide sales	Percent change
1	Humira^®^ (adalimumab)	mAb	TNF (TNF-α)	Abbvie	RA, psoriasis, IBD, others	$14,012 M	$16,078 M	+14.7%
2	Harvoni^®^ (ledipasvir/sofosbuvir)	SM	HCV NS5B polymerase, NS5A	Gilead Sciences	HCV infection	$13,864 M	$9,081 M	−34.5%
3	Enbrel^®^ (etanercept)	Fc fusion	TNF (TNF-α)	Amgen, Pfizer	RA, psoriasis, others	$8,697 M	$8,874 M	+2.0%
4	Rituxan^®^ (rituximab)	mAb	MS4A1 (CD20)	Roche, Biogen	B cell malignancies, RA	$8,354 M	$8,583 M	+2.7%
5	Remicade^®^ (infliximab)	mAb	TNF (TNF-α)	J&J, Merck	RA, psoriasis, IBD, others	$8,760 M	$6,561 M	−10.6%
6	Revlimid^®^ (lenalidomide)	SM	CRBN (E3 ligase cereblon), IKZF1, IKZF3	Celgene	Multiple myeloma	$5,801 M	$6,974 M	+20.2%
7	Avastin^®^ (bevacizumab)	mAb	VEGF	Roche	MCRC, MRCC, others	$6,654 M	$6,752 M	+1.5%
8	Herceptin^®^ (trastuzumab)	mAb	ERBB2 (HER2)	Roche	HER2^+^ breast cancer, gastric cancer, others	$6,509 M	$6,751 M	+3.7%
9	Lantus^®^ (insulin glargine)	Protein	INSR (insulin receptor)	Sanofi	T1D, T2D	$6,770 M	$6,054 M	−10.6%
10	Prevnar 13^®^ (pneumococcal 13-valent conjugate vaccine; CRM197)	Vaccine (conjugated)	Pneumococcal polysaccharides	Pfizer	Pneumonia prophylaxis	$6,245 M	$5,718 M	−8.4%

Abbreviations: CRM197, non-toxic mutant form of diphtheria toxin; mAb, monoclonal antibody; SM, small molecule; J&J, Johnson & Johnson; RA, rheumatoid arthritis; IBD, intestinal bowel disease; HCV, hepatitis C virus; MCRC, metastatic colorectal cancer; MRCC, metastatic renal cell carcinoma; HER2, human epidermal growth factor receptor-2; T1D, type 1 diabetes; T2D, type 2 diabetes

* Data abstracted from La Merie, 2017

** Names given as HUGO Gene Nomenclature Committee (HGNC) names (Gray et al., 2015) followed by commonly used names in parentheses

To date, 74 unique, innovative antibodies and Fc fusion proteins have been approved for treatment of diseases in at least one major market (i.e., US, EU, Japan) (Table 1). Of these, seven have been withdrawn from marketing either due to lack of efficacy, poor toxicity to efficacy profiles, or lack of market interest (Table 1). Of the 74 approved antibody-based molecules, five contain completely murine sequences, nine are mouse-human chimeric antibodies, 26 are humanized, 23 are human antibodies, and 11 are Fc fusions (Table 1). Of the 23 fully human antibodies, 17 are derived from transgenic “humanized” mice and six are derived from human antibody phage display libraries (Table 1). Eight of the Fc fusions are Fc-protein fusions, two are Fc-peptide fusions, and one is an Fc-protein fusion with a tissue-targeting peptide fused to it.

**Figure 1:**
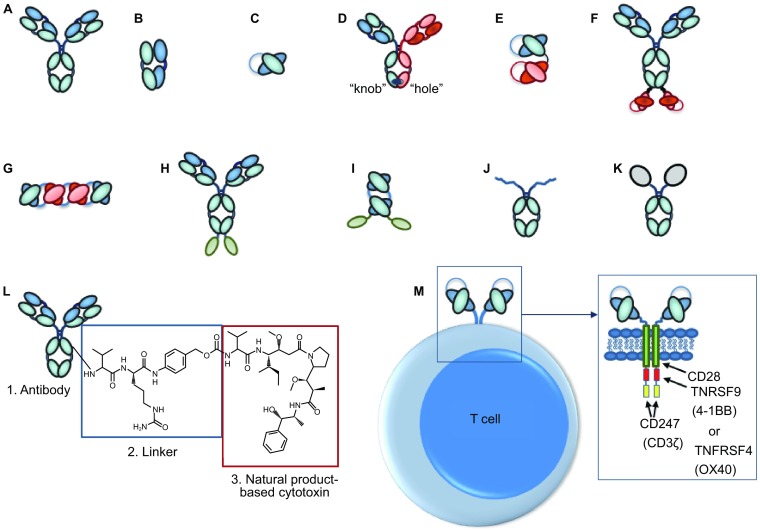
**Cartoons of molecules and constructs discussed**. (A) IgG monoclonal antibody; (B) FAb fragment; (C) Single chain fragment, variable (scFv); (D) Heterodimeric IgG-based bivalent, bispecific antibody; (E) scFv-based bispecific antibody such as a BiTE (“bispecific T-cell engager”); (F) IgG-scFv-based tetravalent, bispecific antibody; (G) Tetravalent scFv-based antibody called TandAb; (H) IgG-based Immunocytokine (cytokine is denoted by green oval); (I) Tandem scFv-immunocytokine (cytokine is denoted by green oval); (J) Fc-peptide fusion (peptides denoted by squiggled lines); (K) Fc-protein fusion (protein denoted by gray oval); (L) Antibody drug conjugate with three parts (antibody, linker, cytotoxic drug); (M) Chimeric antigen receptor (CAR)-T based antibody (scFvs on surface of recombinant T cell; examples of intracellular domains noted in box)

Currently, there are 70 phase III clinical stage candidates, as well as 575 known phase I or phase II antibody-based clinical candidates (Table 3). Thus, as of May 1, 2017, there are at least 719 known antibody and Fc fusion protein clinical-stage candidates (Table 3). Of these, 493 are “naked” IgGs, 13 are “naked” antibody fragments (in both cases, “naked” refers to antibodies that are not antibody-drug conjugates [ADCs], bispecific antibodies, radioimmunotherapeutics, or immunocytokines), 87 are ADCs, 61 are bispecific antibodies, 37 are Fc fusion proteins, 17 are conjugated with radioisotopes either as therapeutics or imaging agents, and 11 are immunocytokines (Table 3 and Fig. 1). It is notable that, with the exception of Fc fusion proteins, most of the non-“naked” antibodies are skewed towards the phase I/II clinical stages, likely due to the more recent development of the various innovative technologies incorporated into those molecules (Table 3).

**Table 3:** Current status of innovative antibody, Fc fusion protein, and chimeric antigen receptor (CAR) drug candidates*

Antibody format	Stage of development			Totals
	Phase I/II	Phase III	Approved for marketing at some point**	
Naked IgG	30	51	52	493
Naked antibody fragments	7	2	4	13
Immunocytokines	9	2	0	11
Fc fusion proteins	23	3	11	37
Bispecific antibodies	58	1	2	61
• IgG-like	• (41)	• (1)	• (1)	• (43)
• Fragment-based	• (14)	• (0)	• (1)	• (15)
• Nanoparticle***	• (03)	• (0)	• (0)	• (03)
Antibody-drug conjugates#	75	9	3	87
Radioimmunoglobulins	13	2	2	17
Antibodies only	575	70	74	719
T or NK cells expressing CAR antibodies	145	0	0	145
Totals	720	70	74	864

Abbreviations: IgG, immunoglobulin G; CAR, chimeric antigen receptor

* From BiStro Biotech Consulting database on clinical stage biologics. Database lock for these data was April 30, 2017

** Innovative antibodies and Fc fusion proteins approved for marketing in a major market (US, EU, Japan)

Five (Raptiva®, 2009; Mylotarg®, 2010; Orthoclone OKT3®, 2011; Bexxar®, 2014; Removab®, 2017) have been withdrawn from marketing, and two others were withdrawn and subsequently were re-approved for new indications under different trade names

*** Bispecific EGFR x *Escherichia coli O*-polysaccharide tandem single chain, Fragment variable (scFv) antibodies that target minicell-derived nanoparticles to tumors

# The 87 antibody-drug conjugates are comprised of 68 small molecule cytotoxic drugs, 10 proteins, and 9 not described

In addition to these protein antibody-derived clinical stage molecules, there are 145 documented phase I or II clinical stage chimeric antigen receptor (CAR)-T cell or natural killer (NK) cell candidates that incorporate antibodies as their CARs (Table 3 and Fig. 1). Thus, there are at least 864 protein and cell based antibody-derived constructs either approved for medical use or being evaluated for their safety and efficacy in clinical trials.

## ANTIBODY TARGETS

The 864 unique antibody-based molecules/cells in development or approved for therapeutic use (Table 3) target 328 unique antigens (Table 4). Because several targets are important for multiple disease areas (e.g., vascular endothelial growth factor [VEGF] as a significant target in both oncology and ophthalmology indications), there are more uses listed than antibodies. Thus, 864 unique molecules are used in 884 different major therapeutic area indications (Table 4), and the 328 unique targets are distributed amongst 351 major uses (Table 5).

**Table 4:** Therapeutic areas targeted by innovative antibodies, Fc fusion proteins, and CARs in clinical development*

Therapeutic area	Major indications for antibodies in phase of development			Totals
	Phases I and II	Phase III	Marketed	
Oncology (antibodies and Fc fusion proteins)	346	30	33	409 (46%)
Oncology (CAR-T and CAR-NK clinical candidates incorporating antibodies)	145	0	0	145 (16%)
Inflammation and autoimmune diseases	132	15	25	172 (19%)
Ophthalmology	16	2	2	20 (2.3%)
Infectious diseases	28	6	4	38 (4.3%)
Neurobiology diseases	20	3	3	26 (2.9%)
Cardiovascular and metabolic diseases	23	0	5	28 (3.2%)
Blood diseases	12	5	4	21 (2.4%)
Pain	3	6	0	9 (1.0%)
Bone and muscle diseases	7	4	2	13 (1.5%)
Other or not disclosed	2	1	0	3 (na)
Total number of uses in each therapeutic area	734	72	78 (all are mAbs/Fc fusion proteins)	884 total uses in therapeutic areas
Total number of unique targets (all therapeutic areas)	--	--	--	328 unique targets
Number of programs per target	--	--	--	Average ~2.7 clinical programs/target

Abbreviations: CAR, chimeric antigen receptor; NK, natural killer; mAbs, monoclonal antibodies; Fc, fragment, crystallizable

* Database lock for these data was April 30, 2017; BiStro Biotech Consulting LLC database. The total number of therapeutic area indications is greater than the number of molecules because some targeted antibodies have been used widely in different indications (e.g., anti-vascular endothelial growth factor [VEGF] antibodies used in both oncology and ophthalmology indications)

**Table 5:** Distribution of targets for antibodies and Fc fusion proteins by major indications in therapeutic areas and location*

Therapeutic area	Antibodies binding to target types				Totals
	Cell-bound targets**	Soluble targets	GPCRs or multi-pass receptors on cells	Infectious agents and toxins	
Oncology	143	26	5	1	175
Inflammation and autoimmune diseases (including asthma, but excluding MS)	52	42	1	0	95
Ophthalmological diseases	1	7	0	0	8
Infectious diseases	2	0	1	11 infectious agents; 3 toxins	17
Neurobiology diseases including MS	7	5	0	0	12
Cardiovascular and metabolism	9	1	4	0	14
Pain and migraine	3	2	1	0	6
Blood homeostasis	3	17	0	0	20
Bone and muscle	2	2	0	0	4
Totals and percent of total	222 (~63%)	102 (~29%)	12 (~3.4%)	15 (~4.3%)	351

Abbreviations: Fc, fragment crystallizable; MS, multiple sclerosis; GPCR, G-protein coupled receptor

* These numbers add up to more than the 328 unique targets noted in Table 4 because several targets have major indications in multiple therapeutic areas (e.g., anti-vascular endothelial growth factor [VEGF] antibodies with major indications in both oncology and ophthalmology)

** Mostly single-pass membrane targets, either as monomeric cell-bound proteins, homodimeric receptors, or heterodimeric receptors

About 62% of these protein and recombinant cell-based candidates are directed against targets in oncology (Table 4). Not surprisingly, all 145 of the current CAR-T and CAR-NK candidates are in clinical evaluation for cancer indications. There are, however, preclinical efforts to generate CAR-T cells against viruses and virus-infected cell targets (Sahu et al., 2013; Liu et al., 2015; Hale et al., 2017), so this may change in the near future. Another 19% of the clinical candidates are directed against targets in the immunology therapeutic area (including autoimmune and asthma, but excluding MS) (Table 4). The remaining ca. 19% of antibody-based proteins are divided amongst other therapeutic areas, including cardiovascular and metabolism, neurobiology, bone and muscle disorders, blood disorders, and infectious diseases.

Of the 351 different uses for targets, 222 (~63%) are single-pass membrane bound proteins or cell-bound proteins (e.g., ERBB2 [erb-b2 receptor tyrosine kinase 2; aka Her2], EGFR [epithelial growth factor receptor], ERBB3 [erb-b3 receptor tyrosine kinase 3; aka Her3], MS4A1 [CD20]). Another 12 (~3.4%) are G-coupled protein receptors (GPCRs; e.g., CCR4 [C-C motif chemokine receptor 4], CCR5 [C-C motif chemokine receptor 5], CXCR4 [C-X-C motif chemokine receptor 4]) or other multi-pass (e.g., CD47, STEAP [six-transmembrane epithelial antigen of the prostate] family members) cell surface targets. Additionally, 102 (~29%) are soluble targets (e.g., TNF [tumor necrosis factor-alpha, TNF-α], IL6 [interleukin-6, IL-6], VEGFA [vascular endothelial growth factor A]), and 15 (~4.3%) are infectious disease targets (e.g., respiratory syncytial virus [RSV]-F protein, *Bacillus anthracis* protective antigen [PA] toxin component, influenza hemagglutinin 2 [HA2; stalk portion], human immunodeficiency virus [HIV] envelop protein gp120) (Table 5).

Cell surface targets in oncology tend to fall into three categories. The first category, which includes about 90 receptors (e.g., CD19, CD20, EPCAM [epithelial cell adhesion molecule, EpCAM], CEACAM5 [carcinoembryonic antigen related cell adhesion molecule 5], MUC1 [mucin 1, cell surface associated]), are essentially “postal addresses” to which killing mechanisms can be targeted directly. These killing mechanisms can include, either individually or in combinations, antibody-dependent cellular cytotoxicity (ADCC) (Ochoa et al., 2017), antibody-dependent cellular phagocytosis (ADCP) (Shi et al., 2015), complement-dependent cytotoxicity (CDC) (Taylor and Lindorfer, 2016), antibody-drug conjugates (ADC) (Tsuchikama and An, 2016; Beck et al., 2017), antibody-induced apoptosis (Sun et al., 2017; Wang et al., 2017), antibody-induced, non-apoptotic programmed cell death (Alduaij et al., 2011), bispecific antibody-redirected killer T or NK cells (Lum and Thakur, 2011; Satta et al., 2013; Suzuki et al., 2015), or CAR-T/CAR-NK cells (Ruella and Gill, 2015; Ruella and June, 2016; Smith et al., 2016). The second group, which overlaps with the first group, are receptors which may be targeted to block ligand binding and signal transduction (Esparis-Ogando et al., 2016; Zhang and Zhang, 2016). The final category are checkpoint modulators, either to block T cell inhibitory pathways or to directly stimulate T or NK cells or macrophages. There are about 20 T-cell related oncology targets in this category.

Of the 328 unique targets for antibody-based drug candidates, the most widely targeted antigen is CD19, which is recognized by 64 clinical candidates, 53 of which are CARs (Table 6). The second most targeted protein is CD3E, found in 32 clinical stage or approved molecules, of which 26 are T cell-redirecting bispecific antibody candidates (Table 6). Thus, the two top targets, CD19 and CD3E, are responsible for the engineered retargeting of T cells, either as CAR-T cells (Ruella and Gill, 2015; Ruella and June, 2016; Smith et al., 2016) or T-cell redirecting bispecific antibodies (Lum and Thakur, 2011; Satta et al., 2013; Suzuki et al., 2015), to kill cancer cells. Of the non-T-cell related targets, the proteins currently most widely targeted are ERBB2 (HER2), EGFR, MS4A1 (CD20), CD22, PDCD1 (PD-1), MSLN (mesothelin), and ERBB3 (Her3), all for cancer indications. The Th17 cytokine, IL17A, to which 14 antibody-related biologics are directed, is currently the top non-oncology target (Table 6). There are 382 unique molecules or recombinant CARs directed against the top 29 targets shown in Table 6, representing about 44% of all of the clinical stage or approved antibody-based molecules/cells; the remaining 482 (~56%) candidates target the remaining 299 unique targets.

**Table 6:** Top targets based on number of molecules developed towards them

Target (alone or in bispecific pairing)	Therapeutic area	Phase of development			
		Phase I/II	Phase III	Approved	Total
CD19	ONC	YYAAAABBB(53T)	Y	B	64
CD3E	IMM, ONC, CVM	YYYYM (24B)	-	YBB	32
ERBB2 (HER2)	ONC	YYYAAABBBBBBB TTTTTTT	Y	YYA	24
EGFR	ONC	YYYATBBBBBBBBB	YY	YYY	19
MS4A1 (CD20)	ONC	YYACCBBTT	YY	YYYRR	16
IL17A	IMM	YYYYYYBBBBB	-	YYY	14
CD22	ONC	ABTTTTTTT	YAAR	-	13
ERBB3 (HER3)	ONC	YYYYYYYYABBB	Y	-	13
PDCD1 (PD-1)	ONC	YYYYYYYYYF	-	YY	12
MSLN (Mesothelin)	ONC	YAAATTTTTTTT			12
APP (Amyloid-β)	NS	YYYYYYYYF	YYY	-	12
VEGFA	ONC, OPHT	YYBBBBB	Y	YYF	11
GD2 ganglioside	ONC	BCTTTTTTTT	-	Y	11
TNF (TNF-α)	IMM	YYYBBF	-	YYYYF	11
CD274 (PD-L1)	ONC	YYYYYYB	Y	YY	10
IL3RA (CD123)	ONC	YYABBBTTT			9
CD33	ONC	YABRTTT	A	A	9
MET (cMet)	ONC	YYYYABBT	Y	-	9
TNFRSF4 (OX40; agonist)	ONC	YYYYYYYF			8
IL6	ONC	YYYYF	-	YYY	8
GPC3 (Glypican-3)	ONC	YBTTTTTT			8
TNFRSF8 (CD30)	ONC	BTTTTTT	-	A	8
CEA	ONC	BBBCRRTT			8
TNFRSF18 (GITR; agonist)	ONC	YYYYYYY			7
EGFR-variant III (EGFRvIII)	ONC	YAATTTT	-	-	7
CD40 (antagonist)	ONC, IMM	YYYYYYY			7
ANGPT2	ONC, OPHT	YYYYBBB			7
IL13	IMM	YYYYB	YY	-	7
FOLH1 (PSMA)	ONC	AABBTTT			7

Abbreviations: A, antibody drug conjugate (ADC); ANGPT2, angiopoietin 2; B, bispecific antibody; C, immunocytokine; F, Fc fusion protein; R, radioimmunoconjugate; IMM, immunology; ONC, oncology; OPHT, ophthalmology; NS, neurosciences; CEA, carcinoembryonic antigen; CVM, cardiovascular/metabolism; FOLH1, folate hydrolase 1; GITR, glucocorticoid-induced TNFR family related gene; PSMA, prostate specific membrane antigen; T, CAR-T, TCR-T, or CAR-NK cells; Y, naked IgG or antibody fragment; other abbreviations are as in Table 1

* Where possible, names given as HUGO Gene Nomenclature Committee (HGNC) names (Gray et al., 2015) followed by commonly used names in parentheses

The 74 approved mAbs and Fc fusion proteins are directed against 39 unique targets, with TNF (TNF-α) and MS4A1 (CD20) being the most widely targeted, with five antibody-based molecules each (Table 1). The five most valuable targets for approved mAbs and Fc fusion proteins are TNF (TNF-α), VEGF, ERBB2 (HER2), MS4A1 (CD20), and PDCD1 (PD-1) (Table 7). Antibodies against the first four of these targets were approved more than ten years ago, so the market value has built up over time. Remarkably, however, the anti-PD-1 antibodies, Keytruda® and Opdivo®, were approved 2014, making PDCD1 (PD-1) a very fast rising target of value (Table 7). The top ten antibody-based therapeutic targets (Table 7) comprise 85% of the value of the total 39 targets, with the anti-TNF molecules leading the way with a market share of 36% (Table 7).

**Table 7:** Most valuable targets for Mabs and Fc fusion proteins as of full-year 2016

#	Target*	Number of drugs	Therapeutic area	Example drugs	First approval of target	Total value 2016**	Percent of total value
1	TNF (TNF-α)	5	Inflammation and autoimmunity	Humira^®^, Enbrel^®^, Remicade®	1998	$38.7 B	36%
2	VEGF	3	Cancer, ophthalmology	Avastin®, Eylea^®^, Lucentis®	2004	$15.3B	14%
3	ERBB2 (HER2)	3	Cancer	Herceptin®, Perjeta^®^, Kadcyla®	1998	$9.5 B	9%
4	MS4A1 (CD20)	3	Cancer	Rituxan®, Gazyva^®^	1997	$7.5 B	7%
5	PDCD1 (PD-1)	2	Cancer	Opdivo®, Keytruda^®^	2014	$6.0 B	5.6%
6	IL12B (p40 subunit of IL-12 and IL-23)	1	Inflammation and autoimmunity	Stelara^®^	2009	$3.2 B (3.23)	3%
7	TNFSF11 (RANK-ligand)	1	Osteoporosis, cancer	Prolia®/Xgeva^®^	2010	$3.2 B (3.16)	3%
8	C5	1	Blood homeostasis	Solira®	2007	$2.8 B	~3%
9	EGFR	3	Cancer	Erbitux^®^, Vectibix^®^, Portrazza^®^	2004	$2.4 B	2.2%
10	IGES (IgE)	1	Asthma	Xolair^®^	2003	$2.3 B	2.2%
Total	--	57	6 different disease areas	--	1997–2014	$90.9 B***	85%
Total market value for all innovator antibodies in 2016						$106.9 B	

Abbreviations: CD, cluster of differentiation; EGFR, epidermal growth factor receptor; HER2, human epidermal growth factor receptor-2; Ig, immunoglobulin; IL, interleukin; PD-1, programmed cell death protein-1; RANK, receptor activator of nuclear factor kappa-B; TNF, tissue necrosis factor; VEGF, vascular endothelial growth factor

* Names given as HUGO Gene Nomenclature Committee (HGNC) names (Gray et al., 2015) followed by commonly used names in parentheses

** Rounded to one decimal point. Data abstracted from La Merie, 2017

*** $90.9 B of $106.9 B is 85% of total mAb and Fc fusion protein value in 2016 (10 of 69 total actively marketed antibody-based products)

**Figure 2:**
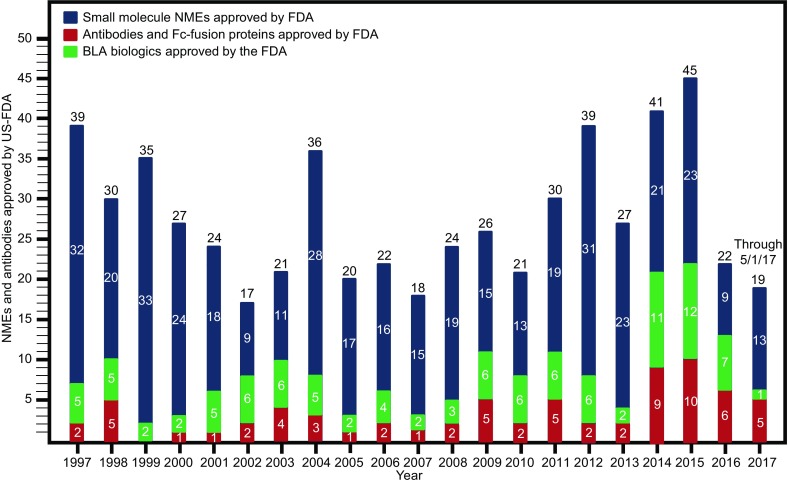
**Small molecule drugs (blue lines), biologics including vaccines (green lines), and monoclonal antibodies/Fc fusion proteins (red lines) approved by the United States Food and Drug Administration from 1997 to May 1, 2017**. This information was sourced and extracted from the US FDA website (https://www.fda.gov/drugs/developmentapprovalprocess/druginnovation/ucm537040.htm)

Based on 2016 sales figures, recombinant proteins comprised seven of the top 10 best selling drugs worldwide (Table 2). Of these seven proteins, five (Humira®, Rituxan®, Remicade®, Avastin®, Herceptin®) are mAbs and one (Enbrel®) is an Fc fusion protein (Table 2). Finally, since January 2014 (the past 3.3 years), antibodies and Fc fusion proteins have comprised 24% (29/121) of innovative United States Food and Drug Administration (US FDA) drug approvals (Fig. 2). This represents the greatest percentage ever since the beginning of the antibody era. Thus, it is clear that mAbs and Fc fusion proteins are making an enormous impact on the pharmaceutical industry, both as novel approaches to treat difficult diseases and meet unmet medical needs, as well as providing an exciting new growth area for the industry.

## BRIEF OVERVIEW OF ANTIBODY ENGINEERING

Human IgGs have been engineered in a multitude of ways to generate different effects (Strohl and Strohl, 2012), as shown in Table 8. In the earlier days of antibody engineering, the focus was on manipulation of the variable regions to humanize and affinity-mature antibodies, or to generate different types of antibody fragments such as scFvs (Bird et al., 1988; Huston et al., 1988), diabodies (Holliger et al., 1993), PEGylated Fabs (Choy et al., 2002), TandAbs (Kipriyanov et al., 1999), and domain antibodies (Ward et al., 1989). The next wave of antibody engineering focused more on the generation and application of “fit for purpose” antibodies (Strohl, 2011) with tuned Fc functions such as increased ADCC, ADCP, and CDC (Strohl and Strohl, 2012; Brezski and Georgiou, 2016; Sondermann and Szymkowski, 2016; Barnhart and Quigley, 2017), or muted or silenced Fc functions (Labrijn et al., 2008; Vafa et al., 2013; Schlothauer et al., 2016; Lo et al., 2017; Borrok et al., 2017). These functions have been approached using both glyco-engineering strategies such as low or no fucosylation for higher FcγRIIIa binding and increased ADCC (Shields et al., 2002; Ferrara et al., 2006; Malphettes et al., 2010; Golay et al., 2013), higher levels of sialylation for dampened immune responses (Anthony and Ravetch, 2010), or non-glycosylated antibodies for partly subdued Fc effector function (Walker et al., 1989; Nesspor et al., 2012). These and more Fc effector modulations can also be generated with amino acid changes in the lower hinge and Fc (Shields et al., 2001; Lazar et al., 2006; Strohl, 2009; Strohl and Strohl, 2012; Vafa et al., 2013; Brezski and Georgiou, 2016; Sondermann and Szymkowski, 2016; Barnhart and Quigley, 2017).

**Table 8:** Examples of antibody engineering and key early dates for the various technologies developed

Type of engineering	Key early date	Notes or comments	Example references
Chimerization	1984	Mouse variable sequences fused to human constant sequences	Morrison et al., 1984
Humanization	1986	Mouse CDRs in human frameworks	Jones et al., 1986; Queen et al., 1989
Generation of scFvs	1989	Fv domains fused with linker	Bird et al., 1988; Huston et al., 1988
Fc fusion proteins	1989	IgG Fc fused with peptides or proteins	Capon et al., 1989
Affinity maturation	1990–1992	Improvement in binding to target	Hawkins et al., 1992
Isotype switching for modified Fc functionality	1990–1993	Change in Fc activity	Greenwood et al., 1993
Aglycosyl IgG	1993	N297x mutation to generate aglycosylated IgG to reduce FcγR activity	Bolt et al., 1993; Nesspor et al., 2012
Heterodimeric Fc engineering to make bispecifics	1996	Knobs-into-holes was first heterodimeric Fc platform	Ridgeway et al., 1996
Silenced Fc activity	1997	IgGσ and other platforms; Abatacept and Eculizumab first clinical candidates to incorporate	Mueller et al., 1997; Vafa et al., 2013
Glyco-engineered for increased ADCC	1999	Increased binding to human FcγRIIIa to increase ADCC; Poteligent®, GlycoMax®; Mogamulizumab and Obinutuzumab first clinical candidates to incorporate	Umana et al.,^1999^; Shields et al., 2002
Modification of protein A binding for purification	2000	First engineering to modulate purification	Tustian et al., 2016
Antibody-cytokine fusions	2001	Cytokine fused to targeting IgG or scFv	Penichet and Morrison, 2001; Halin et al., 2002
Sequence modification for increased Fc activity	2001	Increased binding to multiple FcγRs to increase ADCC, CDC, and/or ADCP	Shields et al., 2001; Lazar et al., 2006
Longer half-life	2002	Modification of Fc sequences to improve pH-dependent binding to FcRn; “YTE” most widely recognized half-life extension modifications	Dall’Aqua et al., 2002; Dall’Aqua et al., 2006
Targeting peptide	2004	RGD targeting of IgG; Asfotase alfa first clinical candidat to incorporate	Li et al., 2004
pH dependent binding to antigen	2010	Improved removal of soluble antigens while recycling antibody	Igawa et al., 2010a; Chaparro-Rogers et al., 2012; Devanaboyina et al., 2013
Modification of pI in variable regions for longer half-life	2010	Engineering variable sequences to improve half-life	Igawa et al., 2010b
Protease-activated “probody” IgG for tumor localized activity	2012	Lack of binding activity until activated by proteolytic cleavage	Erster et al., 2012; Devanaboyina et al., 2013
Clinical candidates using IgG-mediated transcytosis	2012, 2014	Anti-insulin IgG-enzyme fusion for next generation enzyme replacements for CNS ERTs	Boado etal., 2012; Boado et al., 2014
Protease-resistant IgGs	2013	IgG resistant to microbial and tumor-elicited proteases such as MMP9	Kinder et al., 2013
Modification of pI in variable regions for easier purification	2013	Engineering variable sequences to improve purification	Sampei et al., 2013
Sweeping antibodies	2013	Highly active removal of soluble antigens while recycling antibody	Igawa et al., 2013; Igawa et al., 2016
Antibody engineering for improved manufacturability	2014	Modification of variable sequences to improve solubility and decrease aggregation	Clark et al., 2014; Seeliger et al., 2015
Intracellular delivery of IgG	2014	Bioactive IgG escapes endosome to bind to cytosolic target	Choi et al., 2014; Kim et al., 2016
Hexameric IgG formation	2016	Hexamerization of IgGs on cell surfaces with highly improved C1q binding; CDC	Cook et al., 2016; de Jong et al., 2016

Abbreviations: BBB, blood brain barrier; CDC, complement-dependent cytotoxicity; CDRs, complementarity determining regions; CNS, central nervous system; ERT, enzyme replacement therapy

There are currently three approved antibody-based molecules with modified Fc functionality. These include the CTLA4-Fc fusion proteins, abatacept (Orencia®) and belatacept (Nujolix®), both of which have modified hinges to reduce Fc functionality (Davis et al., 2007), and the humanized anti-C5 mAb, eculizumab (Soliris®), which has an IgG2/IgG4 hybrid Fc to reduce Fc functionality (Rother et al., 2007). All three of these molecules bind to immune system components and the muted Fc design was intended to increase the safety margin.

Additionally, two glyco-engineered antibodies with improved ADCC activities have been approved in at least one major market. The first, mogamulizumab (Poteligeo®), is an afucosylated anti-CCR4 mAb approved in Japan for adult T-cell leukemia/lymphoma that is produced by a cell line with a mutation in the *FUT8* (α-1,6-fucosyltransferase) gene (Potelligent® technology from BioWa; Yamane-Ohnuki et al., 2004; Kanda et al., 2006; Malphettes et al., 2010). The second, obinutuzumab (Gazyva®), an anti-MS4A1 (CD20) mAb with low fucose content, has been approved for treatment of chronic lymphocytic leukemia (CLL) (Golay et al, 2013). The low fucose of obinutuzumab is due to the addition to the producing cell line of a β-1,4-N-acetylglucosaminyl-transferase III (GntIII) gene which adds the bisecting N- acetylglucosamine (GlcNAc) that interferes with fucosylation (Glycart GlycoMab® technology acquired by Roche in 2005) (Ferrara et al., 2006). Moreover, there are four glycoengineered (low or no fucose) and one aglycosyl-IgG phase III clinical candidates in the late stage clinical pipeline.

Currently there are no approved IgGs with amino acid-modified increased Fc effector function, although there are two such Fc-modified, increased Fc effector function IgGs in late stage clinical trials, the anti-CD19 mAb, Mor208 (Morphosys, Xencor), in phase II/III clinical trials for treatment of B cell malignancies (NCT02763319), and the anti-ERBB2 (HER2) mAb, margetuximab (Merck, Macrogenics), in phase III clinical trials for breast cancer (NCT02492711).

Moreover, there have been many efforts to modulate aspects of IgG biology beyond just increasing or decreasing Fc effector function (Table 8). The first of these is modulation of half-life via modified interaction of the Fc with the recycling receptor FcRn (Roopenian and Akilesh, 2007; Baker et al., 2009). The most important of these modifications has been the “YTE” mutation (Dall’Aqua et al., 2002; Dall’Aqua et al., 2006) from MedImmune (AstraZeneca), which has been incorporated into a few early stage candidates at this point (Robbie et al., 2013). Other half-life extension mutations of the Fc also have been made, including Xencor’s Xtend technology (Zalevsky et al., 2010), which has been incorporated into at least two early stage clinical candidates, Alexion’s anti-C5 mAb, ALXN5500, and the National Institutes of Health’s (NIH’s) anti-CD4 mAb, VRC01LS.

Several other IgG engineering technologies have been reported that have the potential to modulate the capabilities of existing and future clinical candidates (Table 8). These include protease activated “probody” IgGs for tumor-localized activity (Erster et al., 2012; Desnoyers et al., 2013; Polu and Lowman, 2014), protease-resistant IgGs that are stable in the tumor micro-environment (Kinder et al., 2013), hexameric antibodies with high C1q binding and concomitant CDC effector function (Cook et al., 2016; de Jong et al., 2016), pH-dependent binding (Igawa et al., 2010a; Chaparro-Rogers et al., 2012; Devanaboyina et al., 2013) and sweeping antibodies (Igawa et al., 2013) that improve upon the elimination profile for soluble antigens (Igawa et al., 2016), engineering variable regions for improved solubility and developability (Clark et al., 2014; Seeliger et al., 2015), modulation of the pI or charge of the antibody variable sequences for improved half-life (Igawa et al., 2010b; Li et al., 2014; Datta-Mannan et al., 2015) and/or separation and purification (Sampei et al., 2013), and mutation of protein A binding site for improved purification of a heterobispecific IgG (Tustian et al., 2016). Another area of antibody engineering that is starting to see significant activity is the engineering of IgMs as therapeutics, especially where high avidity effects are desired (Chromikova et al., 2015; Wang et al., 2017b). In a recent example, an anti-TNFRSF10B (DR5) IgM demonstrated 10-fold greater avidity and 1000-fold greater killing effect than a similar IgG (Wang et al., 2017b).

## ANTIBODY DRUG CONJUGATES (ADCs)

ADCs target a cytotoxic drug to a tumor to kill cancer cells while lowering the systemic exposure of the active moiety, with the goal of increasing the size of the efficacy/toxicity window of highly toxic anti-tumor drugs (Strohl and Strohl, 2012; Tsuchikama and An, 2016; Beck et al., 2017). ADCs consist of three components, the targeting antibody, the cytotoxic payload, and the linker that couples those two components together (Fig. 1).

With those three components come five considerations for the design and construction of an ADC: First, the targeting antibody must bind to a protein that is found either exclusively on cancer cells or significantly overexpressed on cancer cells as compared with expression on normal tissues. The best targets for ADCs may be oncofetal antigens or targets that may be overexpressed in cancer cells but present in normal tissues at low copy number or in tissues in which the toxicity is tolerable. The cell surface proteins most widely targeted with clinical stage (or approved) ADCs currently are Her2 (five ADCs targeting), CD19 (four ADCs targeting), CD22 (three ADCs targeting), and mesothelin (3 ADCs targeting). CEACAM5, EGFR (wild-type), EGFR (variant III), CD33, and CD70 each have two clinical stage ADCs targeting them. The properties of good ADC targets, as well as descriptions of candidate ADC targets, have been reviewed (Teicher, 2009; Strohl and Strohl, 2012). An interesting strategy being employed by CytomX to increase the tumor specificity of their ADCs is the use of pro-antibodies that possess a peptide sequence covering the paratope, preventing binding to their target until it reaches the tumor microenvironment (TME). Once in the TME, the paratope-shielding peptide is cleaved by matrix metalloproteinases (MMPs), which are in high concentrations in most TMEs, allowing the antibody to bind to targets in that local environment (Desnoyers et al., 2013; Polu and Lowman, 2014). Second, the ADC-directing antibody must be rapidly internalized upon ligation to its targeted receptor. Antibodies that bind cell surface receptors may or may not internalize rapidly, so when isolating the antibody, incorporation of internalization screens into the discovery process is critical (Poul et al., 2000; Zhou et al., 2010). Third, the identity, number, and type of linker attachment sites is a critical issue. In first generation ADCs, the linkers were typically attached to the ɛ-amine of lysine residues (Tsuchikama and An, 2016; Beck et al., 2017). Given that there are about 80 lysine residues in a typical IgG, ten of which can be accessed for chemical coupling (Tsuchikama and An, 2016), the results of such conjugations are highly heterogeneous. Even with optimization, conjugation to lysines results in a drug to antibody ratio (DAR) of about 2–4, with a range of 0–7 (Lazar et al., 2005; Tsuchikama and An, 2016; Beck et al., 2017). There are multiple challenges with heterogeneous ADCs including analytical challenges, batch-to-batch consistencies, the stability of the ADC, and the potential for variable pharmacokinetics if conjugation sites in some antibodies interfere with normal FcRn-mediated recycling (Beck et al., 2017). Site specific conjugation, which has been achieved through a variety of methods and can result in very tight DARs and increased homogeneity (Junutula et al., 2008; Panowski et al., 2014; Perez et al., 2014; Beerli et al., 2015; Ihospice et al., 2015; Siegmund et al., 2016; Thompson et al., 2016; Tsuchikama and An, 2016; Beck et al., 2017), appears to be a significant advancement. New approaches using extension sequences, such as developed by Mersana, can achieve a drug/antibody ratio of 20 (Yurkovetskiy et al., 2015).

Fourth, the stability of the linker can have a huge influence on the efficacy and toxicity of the ADC. In theory, a more stable linker which is only degraded within the lysosome should have the best safety profile. Unfortunately, it is not that simple, as there are cases in which highly stable linkers resulted in safety issues. Some of these may be due to mannose receptor, or potentially also FcγR-mediated binding and internalization of ADCs, which could result in “off-target” toxicity issues (Gorovits and Krinos-Kiorotti, 2013; Beck et al., 2017).

Finally, not all cancer cells within a tumor are target antigen-positive (Singh et al., 2016), thus allowing potential escape of the antigen-negative cells from targeted therapies. It has been demonstrated that membrane permeability of the cytotoxin is a critical factor for potential bystander activity (Li et al., 2016). Thus, design of future ADCs will need to take the chemistry of the resultant ADC into account to optimize bystander effect and efficacy.

There currently are 87 clinical stage ADCs, including three approved ADCs, nine in phase III development, and another 75 in phase I/II clinical development. The three approved ADCs include Mylotarg® (2000, withdrawn in 2010), the CD30-targeting Adcetris®, and the ERBB2 (Her2)-targeting Kadcyla®. These 87 clinical stage ADC molecules are directed against at least 53 different known targets, although a few have not been disclosed, so the actual number may be higher. The most targeted cell surface receptors currently are ERBB2 and CD19 (4 ADCs against each), and CD33, CD22, and MSLN (mesothelin) (3 ADCs against each).

There are 16 known different classes of drugs incorporated into clinical stage ADCs, 11 of which are small molecule classes and five of which are protein-based. The most widely used drug class incorporated into clinical stage ADCs are the auristatins (employed 31 times), followed by the maytansanoids (in 16 ADCs), and benzodiazepines (used in 9 ADCs) (Table 9). Of the biologics, *Pseudomonas exotoxin* PE38 is incorporated into four ADCs (Table 9).

**Table 9:** Classes of drugs currently being employed in antibody drug conjugate candidates*

Class of drug	Drug type	Number of ADCs per phase			Total
		Phase I/II	Phase III	Approved at some point for Marketing*	
Auristatins	SM natural product-derived	29	1	1	31
Maytansanoids	SM natural product-derived	14	1	1	16
Benzodiazepines**	SM natural product-derived	8	1	0	9
*Pseudomonas aeruginosa* exotoxin PE38	Protein toxin-based	2	2	0	4
Calicheamicin***	SM natural product-derived	1	1	1	3
Diphtheria toxin	Protein toxin-based	2	0	0	2
Irinotecans (SN38)	SM natural product-derived	1	1	0	2
Duocarmycin	SM natural product-derived	2	0	0	2
Exatecan	SM natural product-derived	2	0	0	2
*Staphylococcus aureus* enterotoxin A/E-120	Protein toxin-based	0	1	0	1
Doxorubicin	SM natural product-derived	1	0	0	1
Tubulysin	SM natural product-derived	1	0	0	1
Antibacterial antibiotic	SM	1	0	0	1
Shigatoxin	Protein toxin-based	1	0	0	1
Ricin	Protein toxin-based	1	0	0	1
Urease	Enzyme	1	0	0	1
Not disclosed or unknown	NA	9	0	0	9
Totals		76	8	3	87

* From BiStro Biotech Consulting LLC database on clinical stage biologics. Database lock for these data was April 30, 2017

** Including both pyrrolobenzodiazepines and indolobenzodiazepines

*** Mylotarg, which contained a calicheamicin ADC, was withdrawn from marketing in 2010

Even though three ADCs have been approved for therapeutic use, this technology is still relatively early in the developmental cycle and many of the “rules” for optimized ADCs are still being sorted out (Drake and Rabuka, 2015; Beck et al., 2017). More details on the design and construction of ADCs can be found in Tsuchikama and An (2016) and in Beck et al. (2017).

## Fc FUSIONS

Fc fusions are fusions of the IgG Fc domain with either a protein or peptide. In theory, the fusion can be to either the C- or N-terminus of the Fc, but most Fc fusions on the market and in clinical development today are N-terminal fusions. The primary reason for generating Fc fusions is to extend the half-life of pharmacologically relevant protein or peptide by using the FcRn-mediated recycling of the Fc (Strohl and Strohl, 2012; Strohl, 2015). Currently, 11 Fc fusion proteins have been approved for therapeutic use (Table 1), three are in phase III clinical trials, and 23 are being evaluated in earlier stage clinical trials (Table 3). Many of the earlier Fc fusions generally were constructed using receptor exodomains in immune pathways (e.g., TNFRSF18 [p75], CD58 [LFA3], CTLA4, IL1R1 [IL-1 receptor]) fused to the Fc to modulate the immune system, either by blocking soluble cytokines or by binding to cells. More recent Fc fusion proteins have become more diverse (Strohl, 2015), with the pharmacologically active “head groups” being blood factors, such as F9 (Factor IX) and F8 (Factor VIII), peptides such as GCG (GLP-1) and a THPO (thrombopoietin) analogue, and an enzyme, such as the tissue non-specific alkaline phosphatase (TNSALP; Millan et al., 2008) in asfotase alfa (Strensiq®) (Hofman et al., 2016).

## IMMUNOCYTOKINES

Certain human cytokines such as IL2 have been approved (marketed name, Proleukin®) for systemic delivery and use in severe diseases such as metastatic melanoma and metastatic renal cell carcinoma (Dutcher, 2002). Systemic delivery of the T cell-activating cytokine, IL2, however, brings with it the potential for adverse events. The concept of using antibodies to target cytokines to either tumors or to specific tissues came into fruition around the turn of the century (Penichet and Morrison, 2001; Halin et al., 2002). Since that time, there has been an effort to target IL2, or other cytokines such as IL12 and TNF, to the tumor microenvironment, where the desired activity can take place with reduced adverse systemic effects (Neri and Sondel, 2016). This approach has been actualized by the fusion of cytokines to antibodies to make immunocytokines that may target vasculature associated with tumors (Pasche et al., 2012; Hemmerli and Neri, 2014), tumor cell surface antigens (Klein et al., 2017), or targets that would assist in accumulation in inflamed joints (Hughes et al., 2014). Immunocytokines come in two major formats, cytokine-scFv (or other fragment) fusions which have a short circulating half-life and cytokine-IgG fusions, which retain a long half-life (Neri and Sondel 2016).

There are at least 11 immunocytokines currently being evaluated in clinical trials. Two of these are Darleukin® (fibronectin extra domain B [EDB]-targeting scFv L19-IL2 fusion) and Fibromun® (EDB-targeting scFv L19-TNF fusion), which are both in phase III pivotal clinical trials as combination therapy for malignant melanoma (NCT02938299). Other clinical stage immunocytokines include examples such as Dekavil® (fibronectin extra domain A [EDA] targeting scFv F8-IL-10 fusion in phase II for treatment of RA [NCT02270632]), Teleleukin® (tenascin C alternative splice variant EDA1-targeting scFv F16-IL-2 fusion in phase I for treatment of acute myeloid leukemia [AML; NCT02957032]), RG7461 FAP (fibroblast-activation protein)-IL2 fusion in phase I for treatment of solid tumors [NCT02627274]), and cergutuzumab amunaleukin, an anti-CEA (carcinoembryonic antigen-IgG fused with IL2, currently in phase I clinical trials (NCT02350673) for treatment of solid tumors.

## CHECKPOINT MODULATORS

Antibody-directed modulation of immune cell checkpoint receptors has become one of the most exciting and important new areas in antibody therapeutics over the past few years. Most efforts have been focused on T cell checkpoint modulation, but there is increasing interest in B cell, NK cell, and myeloid cell checkpoint modulation as well.

T cell activation is regulated by a series of three signals. The first signal is provided by the interaction of the T cell receptor (TCR) with major histocompatibility complex (MHC, HLA) class I (for CD8 T cells) or MHC (HLA) class II (for CD4 T cells) on antigen presenting cells (APCs). The secondary signal is provided through one of several checkpoint receptors (Table 10), which can either provide a costimulatory signal to activate the T cells, or a blocking signal to dampen T cell response (Topalian et al., 2015). The third signal comes from the production of either pro-inflammatory, T cell-activating cytokines or anti-inflammatory cytokines that would act to reduce T cell response (Chikuma et al., 2017; Schirdewahn et al., 2017).

**Table 10:** mAbs and Fc fusion proteins directed towards immunomodulation and checkpoint targets*

Target***	Activity	Therapeutic area	Phase of development			Total number of candidates
			Phase I/II	Phase III	Approved	
CD80/CD86	Antagonist	IMM	0	0	2	2
CTLA4	Antagonist	ONC	1	1	1	3
PDCD1 (PD-1)	Antagonist	ONC	13	0	2	15
CD274 (PD-L1, B7-H1)	Antagonist	ONC	6	1	3	10
PDCD1LG2 (PD-L2)	Antagonist	ONC	1	0	0	1
CD28	Antagonist	IMM	3	0	0	4 total
	Agonist	ONC	1	0	0	
TNFRSF4 (OX40)	Antagonist	ONC	2	0	0	10 total
	Agonist	ONC	8	0	0	
TNFSF4 (OX40 ligand, CD252)	Antagonist	ONC	0**	0	0	0
CD40	Antagonist	IMM	7	0	0	13 total
	Agonist	ONC	6	0	0	
CD40LG (CD154; CD40 ligand)	Antagonist	ONC	2	0	0	2
ICOS (CD278)	Antagonist	ONC	1	0	0	3 total
	Agonist	ONC	2	0	0	
ICOSLG (ICOS-ligand; B7RP-1; CD275)	Antagonist	IMM	1	0	0	1
TNFRSF18 (GITR)	Agonist	ONC	7	0	0	7
HAVCR2 (TIM3)	Antagonist	ONC	2	0	0	2
TNFRSF9 (CD137, 4-1BB)	Agonist	ONC	2	0	0	2
LAG3 (CD223)	Antagonist	ONC	3	0	0	3
VSIR (VISTA)	Antagonist	ONC	1	0	0	1
TIGIT	Antagonist	ONC	2	0	0	2
CD47	Antagonist	ONC	4	0	0	4
CD27	Agonist	ONC	1	0	0	1
Totals	--	--	76	2	7	85

* Abbreviations: IMM, immunology; ONC, oncology

** Known preclinical programs that should progress to clinical trials by end of 2017

*** Names given as HUGO Gene Nomenclature Committee (HGNC) names (Gray et al., 2015) followed by commonly used names in parentheses

Cancer cells can express ligands for T cell inhibitory receptors such as PDCD1 (PD-1) (ligand is CD274 [PD- L1]), CTLA-4 (ligands are CD80 and CD86), and HAVCR2 (aka TIM3) (ligand reported to be GAL9) to inhibit T cell activation and cytolytic T cell responses. Ligation of these receptors can lead to T cell anergy or exhaustion, resulting in the inability of the immune system to kill cancer cells. Inhibition of the blocking responses to T cell activation using anti-PDCD1, anti-CTLA4, or anti-CD274 antibodies has proven clinically to result in improved responses for a subset of patients with metastatic melanoma, NSCLC, and potentially other forms of cancer (Achkar and Tarhini, 2017; Kim et al., 2017). Additionally, efforts are ongoing to use combinations of anti-PD1 and anti-CTLA4 antibodies to increase the percentage of patients experiencing durable responses, i.e., “raising the tail of the survival curve” (Harris et al., 2016). Alternatively, several clinical candidates are agonists of T-cell activating receptors such as TNFRSF4 (OX40), CD40, TNFRSF9 (CD137, 4-1BB), TNFRSF18 (GITR), ICOS (CD278), CD27, or CD28 to stimulate T cell responses (Antonia et al., 2016; Table 10).

Additionally, T cell checkpoint pathways are potentially important in infectious diseases, in which T cell exhaustion halts T cells from eliminating viral and bacterial pathogens (Dyck and Mills, 2017). Finally, antibody intervention in T cell checkpoint pathways may play a role in autoimmune diseases, where blocking the activating signals or increasing the blocking signals may result in lowering the T cell activation response (van der Vlist et al., 2016).

Five mAbs and two Fc fusion proteins that target T cell/APC checkpoints have been approved (Table 10). Two more T cell checkpoint inhibitor antibodies are currently in phase III clinical trials and 77 are in phase I/II clinical trials, covering 19 different T cell checkpoint targets. Some of these checkpoint targets are being tested in both immune and oncology related diseases. For example, CD28, CD40, and TNFRSF4 (OX40) antagonists are in early stage clinical trials for treatment of various immune disorders, whereas CD28, CD40, and TNFRSF4 (OX40) agonists are in early stage clinical trials for various cancer indications (Table 10).

Checkpoint ligands expressed on cancer cells also are potentially excellent targets, both because they can block the inhibitory checkpoint interaction as well as targeting the ligand-expressing cancer cells with Fc-active antibodies. For this approach, there are now three approved anti-PD-L1 antibodies and another seven in clinical trials, as well as three clinical stage anti-CD70 (CD27 ligand) mAbs and one CD70-targeting CAR-T cell product in phase I clinical trials., as well as four anti-CD276 (B7H3) antibodies are currently in phase I clinical trials.

B cell transitional checkpoints are centered around B cell homeostasis and the choice of whether the B cell should mature or proceed to apoptosis. This process ensures that B cells expressing autoreactive immunoglobulins are purged (Cancro et al., 2009). Key regulators of B cell maturation that function in B cell checkpoints are TNFSF13B (soluble BLyS, ligands B lymphocyte stimulator; also known as B cell activating factor [BAFF]) and TNFSF13 (APRIL, a proliferation-inducing ligand). TNFSF13B can bind the TNFSF13B receptor (BR3; also known as BAFF-R) to promote B cell survival, and both TNFSF13B and TNFSF13 can bind TNFRSF13B (transmembrane activator-1 and calcium modulator and cyclophilin ligand-interactor, TACI) and TNFRSF17 (B cell maturation antigen, BCMA), both of which result in Ig class switching and T cell-dependent responses (Cancro et al., 2009).

Overexpression of TNFSF13B can lead to autoimmune consequences, such as system lupus erythematosus (SLE) or Sjögren’s syndrome (Cancro et al., 2009). One B cell checkpoint inhibitor (anti-TNFSF13B mAb, Benlysta®) is approved, two more are currently in phase III clinical trials, and three are in phase I/II clinical trials, all targeting the B cell activating factor regulatory pathway.

Another approach that has gained interest in very recent years is the immunomodulation of NK cells. NK cells, as well as CD8 T cells, express a series of inhibitory receptors including KLRC1-form A (NKG2A), TIGIT, CD96, and KIR family members (Carotta, 2016). As an immune defense mechanism, tumor cells express ligands to bind to these receptors to inhibit unwanted activation of NK cells. Currently there are six antibodies in phase I/II clinical trials binding these targets to remove the brake on NK cell activation.

Finally, another checkpoint that regulates the activity of macrophages and their phagocytosis of target cells is the CD47/SIRPA (signal regulatory protein alpha) and CALR (calreticulin)/LRP1 pathway. The CD47/SIRPA ligation is often referred to as the “don’t eat me” signal, whereas CALR/LRP1 ligation is known as the “eat me” signal (McCracken et al., 2015). Blocking of CD47 by antibodies or Fc fusion proteins can lead to an imbalance and a pro-“eat me” response (McCracken et al., 2015). Currently, four anti-CD47 antibodies or Fc fusion proteins are being evaluated in clinical trials for treatment of cancer (Table 10).

## ANTIBODY MIXTURES

One approach that has gained interest in recent years is the combination or mixture of antibodies, usually against a single target, included into a single dosage (Raju and Strohl, 2013; Carvalho et al., 2016). Thus far, antibody mixtures are being used mostly for oncology and infectious disease indications. The Danish biotechnology company, Symphogen, has led this space, with four antibody mixtures currently being tested in clinical trials. These include SYM004, a mixture of two anti-EGFR mAbs, SYM013, a mixture of six antibodies against the ERBB (Erb-b2 receptor tyrosine kinase) family of receptors (Ellebaek et al., 2016), SYM015, a mixture of two antibodies targeting MET (cMET), and SYM009, an undisclosed mixture of antibodies partnered with Genentech for an infectious disease target. At least nine other antibody mixtures are being evaluated in clinical trials, all of which are against infectious diseases targets such as Ebola virus, botulinum toxin, and other viruses.

One very interesting new approach in this area that could see significantly greater upside in the coming years is the generation of fully human antibody mixtures, or polyclonal mixtures, in transgenic (tg) cattle (Matsushita et al., 2014, 2015). These may, if found safe and efficacious, at least partially replace “specific” intravenous immunoglobulin (IVIG), which is IgG purified from individuals who have been vaccinated or from convalescing patients who have produced IgGs against a specific target (Llewelyn et al., 1992; Mire et al., 2016). The upside of tg cattle-produced human IgGs is supply, consistency across lots, and the ability to vaccinate the cows with antigens not available for human vaccination due to regulatory and safety considerations. One such polyclonal mixture from tg cattle already being evaluated in clinical trials is SAB-301 (SAB Therapeutics), a polyclonal mixture of human IgGs targeting middle east respiratory (MERS) virus (NCT02788188; Luke et al., 2016).

## BISPECIFIC ANTIBODIES

**Figure 3:**
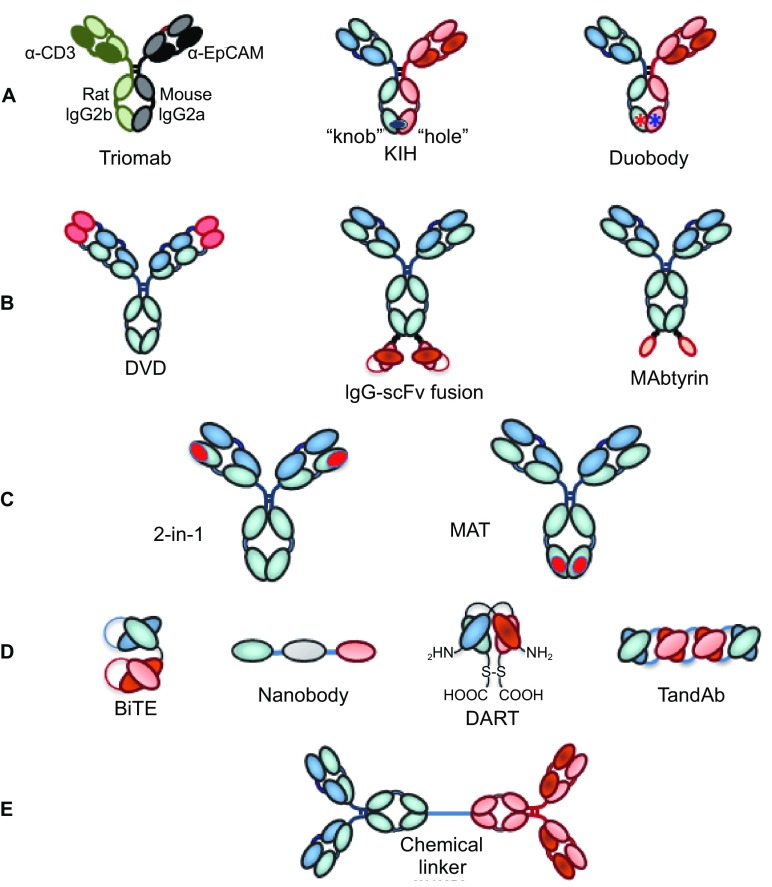
**Five basic types of bispecific antibodies**. (A) Bivalent, bispecific IgG-like antibodies with heteromeric heavy chains (e.g., Triomab, knobs-into-holes (KIH), Duobody, etc); (B) Tetravalent multispecific antibodies comprised of IgGs with other binding domains fused to either the N- or C-termini of either the heavy or light chains (e.g., dual variable domain [DVD], IgG-scFv fusion, Mabtyrin (IgG with non-antibody binding scaffold “centyrin” fused to C-terminal end of heavy chains); (C) IgGs to which additional antigen combining sites have been added within the structure (e.g., two-in-one antibodies, MAT “Modular Antibody Technology” platform from F-Star); (D) Engineered antibody fragments linked by short peptide linkers which can be made into bivalent, trivalent, or tetravalent formats addressing two to three targets (e.g., bispecific T-cell engager (BiTE), Nanobody platform, dual- affinity re-targeting (DART) antibodies, “tandem antibody” structures (TandAbs)); (E) Chemically coupled IgGs

 Bispecific antibodies, first conceptualized in 1983 (Milstein and Cuello, 1983), are antibodies that can bind two different antigens simultaneously. There are five fundamental groups of bispecific antibody formats: (i) asymmetric bivalent, bispecific IgG-like antibodies with heterodimeric heavy chains (HCs) (Ridgeway et al., 1996; Merchant et al., 1998; Gunasekaran et al., 2010; Strop et al., 2012; Klein et al., 2012; Labrijn et al., 2013 Von Kreudenstein et al., 2013; Brinkmann and Kontermann, 2017); (ii) tetravalent multispecific antibodies that are comprised of IgGs, with additional binding domains, e.g., scFvs, Fvs, VHH domains, or non-antibody binding scaffolds such as fynomers (Brack et al., 2014; Silacci et al., 2016), fused to either the N- or C-termini of either the heavy or light chains (LCs) (Coloma and Morrison, 1997); (iii) engineered binding domains within the normal IgG structure, such as the “two-in-one” bispecific approach from Genentech (Bostrom et al., 2009; Eigenbrot and Fuh, 2013) and the F-STAR approach of designing novel second binding sites within the C_H_3 domain (Leung et al., 2015), (iv) engineered antibody fragments linked by short peptide linkers which can be made into bivalent, trivalent, or tetravalent formats addressing two to three targets (Mack et al., 1995; Holliger and Winter, 1997; Kipriyanov et al., 1999; Reusch et al., 2015; Egan et al., 2016). These may be fused to an Fc domain or other half-life extending molecule (Liu et al., 2017); and (v) IgGs that are chemically coupled to generate IgG-IgG conjugates (e.g., Brennan et al., 1985; Garrido et al., 1990). Examples of these five basic formats are shown in Fig. 3. Many variations on these central themes have been reviewed multiple times (Kontermann, 2012; Spiess et al., 2015; Kontermann and Brinkmann, 2015; Ha et al., 2016; Brinkmann and Kontermann, 2017).

Over the past decade there has been a literal explosion of novel bispecific antibody technologies, approaches, and clinical candidates. Today there are at least 61 bispecific or bifunctional antibodies in clinical trials that are made from at least 24 different bispecific platform technologies (Table 11). These include ten asymmetrical IgG-based platforms (17 bispecific antibodies), five appended IgG platforms (17 bispecific antibodies), a single platform for chemically coupled IgGs (four bispecific antibodies), eight fragment-based platforms (22 bispecific antibodies), and one IgG-based bispecific generated with an unknown platform (Table 11). Two bispecific antibodies have thus far been approved for medical use, both in the field of oncology. The first bispecific antibody of any kind to be approved was catumaxomab (Removab®), a bivalent, trifunctional, hybrid mouse IgG2a – rat IgG2b antibody targeting CD3E with one arm and EPCAM with the other. Catumaxomab, approved in 2009 (only in the European Union) for treatment of malignant ascites, was generated by the three-way fusion of a mouse B-cell, a rat B-cell, and a myeloma cell to form a quadroma cell line (Triomab® technology) (Zeidler et al., 1999). The second bispecific antibody to be approved was the anti-CD3E x anti-CD19 “Bispecific T Cell Engager” (BiTE) MT-103, constructed by linking two scFvs with a five residue (G4S)1 linker (Mack et al., 1995). This BiTE®, now known as blinatumomab (trade name, Blincyto®), was approved in 2014 for treatment of B-cell acute lymphoblastic leukemia (ALL).

**Table 11:** Summary of bispecific antibody platforms currently represented by clinical candidate antibodies

Bispecific antibody platform	Subgroup	Group in Fig. 3	Light chain solution	Number of clinical candidate antibodies	Immune cell redirected candidates	Most advanced candidate	Company or institute	Reference for platform
Rat/mouse Triomab	Asymmetric	A	Species-specific LCs	1	1	Approved in EU	Fresenius, Trion	Zeidler et al., 1999
ART-Ig	Asymmetric	A	CLC	1 (+ 1 IgG-scFv*)	0	Phase 3	Chugai	Sampei et al., 2013
Knobs-into-holes	Asymmetric	A	CLC, CFS	5	1	Phase 2	Genentech	Ridgeway et al., 1996; Merchant et al., 1998
Duobody	Asymmetric	A	Post-production assembly	3	1	Phase 1	Genmab	Labrijn et al., 2013
Biclonics	Asymmetric	A	CLC	2	1	Phase 1	Merus	Throsby et al., 2016
BiMab	Asymmetric	A	CLC	1	0	Phase 1	Oncomed	http://drugspider.com/drug/navicixizumab
Azymetric (ZW1)	Asymmetric	A	LC mutations	1	0	Phase 1	Zymeworks	Von Kreudenstein et al., 2013
Xmab HA	Asymmetric	A	Fab plus scFv	1	1	Phase 1	Xencor	Moore et al., 2011
BEAT	Asymmetric	A	Fab plus scFv	1	1	Phase 1	Glenmark	Moretti et al., 2013
Protein A differential	Asymmetric	A	CLC	1	1	Phase 1	Regeneron	Smith et al., 2015; Tustian et al., 2016
IgG-scFv	Appended IgG	B	NA	6*	1*	Phase 2	Eli Lilly, Merrimack, and others	Coloma and Morrison, 1997
DVD-Ig and DVD-Ig-like	Appended IgG	B	NA	3	0	Phase 2	Abbvie	Wu et al., 2007
IgG-Fab	Appended IgG	B	CFS	2	1	Phase 1	Roche	Klein et al., 2016
IgG-peptide	Appended IgG	B	NA	3	0	Phase 1	Medimmune	Konkar et al., 2016
IgG-fusion protein or enzyme	Appended IgG	B	NA	3	0	Phase 1	Roche, Armagen	Boado et al., 2007; 2012; 2014
Chemically coupled IgGs	Chemically coupled IgGs	E	NA	4	4	Phase 2	Barbara Ann Karmanos Cancer Inst	Brennan et al., 1985; Garrido et al., 1990
BiTE	Antibody fragment-based	D	NA	5	5	Approved in US and EU	Amgen (Micromet acquisition)	Mack et al., 1995; Schlereth et al., 2005; Baeuerle et al., 2008
TandAb	Antibody fragment-based	D	NA	2	2	Phase 2	Affimed	Kipriyanov et al., 1999
Tandem scFv	Antibody fragment-based	D	NA	4	0	Phase 2	EngeneIC and several others	Madrenas et al., 2004
Dock-and-Lock	Antibody fragment-based	D	NA	1	0	Phase 2	Immunomedics	Sharkey et al., 2010
DART and DART-Fc	Antibody fragment-based	D	NA	6	5	Phase 1	Macrogenics	Johnson et al., 2010; Root et al., 2016
Nanobody-based	Antibody fragment-based	D	NA	2	0	Phase 1	Ablynx	Van Heusden et al., 2013
TCR-scFv	Antibody fragment-based	D	NA	1	1	Phase 1	Immunocore	Bossi et al., 2014
ADAPTIRTM	Antibody fragment-based	D	NA	1	1	Phase 1	Aptevo	Hernandez-Hoyos et al., 2016
Unknown design	IgG-based	-	unk	1	0	Phase 1	BioCad	(NCT03103451)
Totals	24 different known platforms		--	61 different candidates	27	--	--	--

* One of the ART-Ig molecules is constructed as an IgG-single scFv

Abbreviations: ART-Ig, asymmetric re-engineering technology-immunoglobulin; BEAT, bispecific engagement by antibodies based on the T cell receptor; BiTE, bispecific T cell engagers; DART, dual affinity retargeting (antibody); DVD-Ig, dual variable domain-immunoglobulin; LC, light chain; CLC, common light chain; CFS, cross-Fab (or cross-mAb) sequences; Fab, fragment, antibody; Fc, fragment, crystallizable; scFv, single chain, fragment, variable; LC, light chain; NA, not applicable; scFv, single chain Fragment, variable; TandAb, tandem diabodies; TCR, T cell receptor

The 61 current clinical stage bispecific antibodies are used for a variety of different purposes. For example, 10 of them bind two soluble antigens such as IL13 and IL4 (e.g., SAR156597; NCT02345070), nine bind two receptors on the same cell surface such as EGFR and MET (e.g., JNJ-61186372; NCT02609776), and four bind a cell surface target such as Delta Like Canonical Notch Ligand 4 (DLL4) with one combining site and a soluble ligand such as VEGF with the other (e.g., navicixizumab; NCT02298387). Two current clinical stage bispecific antibodies are biparatopic, i.e., both arms bind to the same receptor, albeit at two different non-overlapping epitopes (e.g., Zymeworks ZW25, which binds two non-overlapping epitopes of ERBB2; NCT02892123).

The most significant use of bispecific antibodies, however, is for T cell redirection, in which one combining site is directed toward a cell surface target on a cancer cell and the other combining site binds CD3E on T cells to redirect those cells to the targeted cancer cell (see below).

## T CELL REDIRECTION

Twenty-seven clinical stage bispecific antibodies are immune cell redirection bispecific antibodies. One of these targets FCGR3A (CD16a) for NK cell redirection, while the other 26 bispecific antibodies target CD3E on T cells to redirect the cytotoxic T cells (CTLs) to kill and lyse cancer cells. Of these, 14 are constructed from antibody fragments, seven are asymmetric bispecific IgGs, four are conjugated bispecific IgGs that are used to activate T cells *ex corporally* (Brennan et al., 1985; Garrido et al., 1990), and two are bispecific IgGs with appended domains (Table 11). The two appended IgGs also utilize an asymmetric Fc format so that only one CD3E-binding arm is present. It is generally accepted that the most potent T cell redirecting antibodies are fragments, with unmodified BiTEs and DARTs (dual-affinity re-targeting antibodies) demonstrating sub-picomolar IC50 values for *in vitro* killing activities (Moore et al., 2011). Of the two approved antibodies, Blincyto® is a mouse BiTE, while Removab® is an asymmetric rat/mouse IgG. Given that both are “first generation” T cell redirecting, fully mouse antibodies for very different indications, it is difficult to say today which type of platform (fragment vs. IgG-based) will ultimately be the most efficacious for treatment of diseases. The larger IgG-based forms appear to be significantly less potent based on *in vitro* activities and *in vivo* preclinical dosing than are fragments (unpublished data). Thus, there is a balance between sheer potency, which can be achieved with small size, and long half-life, which typically brings with it greater size and less potency. Additionally, both the size of the cell surface receptor of the target cells and the epitope to which the antibody binds appear to be critical factors in potency as well (Bluemel et al., 2010). Moreover, the potency of T cell redirected bispecific antibodies depends on the affinity of the arms for each antigen. Typically in the case of bispecific T cell redirection antibodies, the affinity for the cancer cell surface target is much higher (i.e., 10-fold or more) than the affinity for the CD3E chain on T cells (Zhukovsky et al., 2016). In summary, factors that may influence potency in T cell redirected antibodies are size of the antibody, size of the target cell surface protein, epitope on that protein to which the antibody binds, and affinity.

Another area that has not yet been fully investigated with respect to T cell redirection is the role of Fc functionality. The Triomab® platform, on which Removab® is designed, has a highly active Fc domain that interacts with human FcγRs to increase the immune response (Chelius et al., 2010; Hess et al., 2012). On the other hand, most of the current fragment-Fc, asymmetric IgG, or appended IgG platforms have used muted or silenced Fcs so as not to over stimulate the immune system via interactions with myeloid effector cells. Even with the absence of Fc activity, many treatments with T cell redirecting bispecific antibodies are accompanied by cytokine storms that need to be addressed as part of the therapeutic paradigm (Lee et al., 2016). Thus, it seems likely that most T cell redirecting antibodies made in the future will continue to avoid Fc activity in an effort to limit the release of pro-inflammatory cytokines by T cells and other effector cells in the tumor microenvironment.

## CAR-T CELLS AND TCR-T CELLS

CARs are anti-tumor targeted antibodies that have been fused genetically to a stalk or linker, a transmembrane domain, and intracellular T cell activation domains that have been borrowed from activation checkpoint receptors such as CD28, TNFRSF9 (CD137), and/or TNFRSF4 (OX40) (Fig. 1M; Figueroa et al., 2015; van der Stegen et al., 2015; Ruella and Gill, 2015; Smith et al., 2016; Ruella and June, 2016; Lim and June, 2017). While the concept of CAR-T cells has been around since the early 1990s (Eshhar et al., 1993), the advancement of technologies required to turn this into a viable “manufacturable” process was only realized in recent years. Thus, similar to bispecific antibody technology, while conceptually old, truly developable CAR-T technology is still relatively young and still developing (Lim and June, 2017).

There are fundamentally two types of CARs. The first is autologous, in which a patient’s T cells are collected by a process known as apheresis, and then either as a whole pool, or a fractioned pool of CD8 T cells, CD4 T cells or possibly both CD4 and CD8 T cells, are transduced with the CARs using either viral vectors such as Lentivirus or transposons such as Sleeping Beauty or PiggyBack (Figueroa et al., 2015; Lim and June, 2017). The recombinant T cells, now armed with CARs targeting a tumor expressed on their surface, are activated and infused back into the patients from which they were derived to kill cancer cells bearing the antigen (Figueroa et al., 2015).

The second major type of CAR is allogeneic, or universal. An “off-the-shelf” cell line is constructed, typically devoid of MHC class I molecules (Ren et al., 2017a) and endogenous T cell receptors (MacLeod et al., 2017; Ren et al., 2017a) to decrease the risk of host vs. graft (rejection) and graft vs. host disease (GvHD), respectively. This universal T cell line also would express CARs for treatment of cancer or possibly viral infections. Thus far, the barriers to generate truly off the-shelf allogeneic cell lines are still quite high, with control of proliferation, continued activation of the cells once they are engrafted, and incorporation of kill switches for safety purposes as critical issues still to be worked out. Nevertheless, significant progress has been made in just the past year suggesting that fully modified allogeneic CAR-T cell therapy is quickly becoming a reality (Ren et al., 2017a, b). To date, there are four generations of autologous CAR-T cell constructs. The first generation typically consisted of the extracellular, cancer cell-targeting scFv fused to the CD8 stalk and transmembrane domain followed by CD247 (aka CD3ζ), which provided the activation signal (Park and Brentjens, 2010; Figueroa et al., 2015; Lim and June, 2017). The first generation CARs possessed ample cytotoxicity but lacked proliferative and survival signals. The second-generation CARs typically linked the exodomain scFv to the transmembrane domain of CD28, TNFRSF9 (CD137, 4-1BB), or TNFRSF4 (OX40) to provide a proliferation signal, followed by CD247 (CD3ζ) to provide the cytolytic activation signal. The third generation CARs have typically linked the targeting scFv to the CD28 transmembrane domain, followed by either the TNFRSF9 (CD137, 4-1BB), or TNFRSF4 (OX40) activation domains, and then CD247 (CD3ζ) (Park and Brentjens, 2010; Figueroa et al., 2015; Smith et al., 2016; Lim and June, 2017). These CARs combined cytolytic activity with both proliferation and survival signals to enhance both their activity and their persistence in the patient’s serum. Fourth generation CARs add new activities such as a suicide mechanism to kill off the CARs in case they become over-proliferative, or utilize T cells that have been conditioned to recognize viral antigens which can be used as “vaccines” to increase the persistence of the CAR-T construct (Chmielewski et al., 2014; Smith et al., 2016; Lim and June, 2017).

There are currently 145 different CAR constructs in clinical trials. As stated earlier, all of the CAR candidates are in phase I or II clinical trials. Almost half (72/145) of the current CARs originated in China, with 67 originating in the US, and 6 originating in Europe. CARs have been generated against 38 different targets, 37 of which are cell-surface proteins on cancer cells and one, WT1, an MHC-displayed peptide target derived from an intracellular antigen (Rafiq et al., 2017). Fifty-three (~37%) clinical CAR candidates are directed against CD19. The next most targeted antigens are GD2 and MSLN (mesothelin) (8 CARs each), ERBB2 (HER2) and CD22 (7 CARs each), and GPC3 (glypican-3) and TNFRSF8 (CD30) (6 CARs each). Most of the current clinical stage CAR constructs are autologous CAR-T constructs generated from αβ T cells (Table 12), but there are a few examples of other formats, including early formats of allogeneic CAR-T cells, autologous CAR γδ T cells, both autologous and allogeneic CAR-NK cells, CAR-NKT cells, and CARs made from TCRs (Table 12).

**Table 12:** Chimeric antigen receptor (CAR)- and T-cell receptor (TCR)- based immuno-oncology clinical candidates*

Type	Number
Autologous CAR αβ T cells	128
Autologous CAR γδ T cells	1
Allogeneic CAR αβ T cells	6
Autologous CAR-NK cells	3
Allogeneic CAR-NK cells	2
Autologous CAR-NKT cells	2
Autologous recombinant TCR-T cells	3
Total number of CAR and CAR-like clinical candidates	145

* From BiStro Biotech Consulting LLC database on clinical stage biologics. Database lock for these data was April 30, 2017

It is too early to judge the success of the CAR field, although it is clear that this area has generated an enormous amount of interest, as well as funding well exceeding $1B. It is noteworthy that Novartis recently (3/29/17) filed a biologics license application (BLA) to the US FDA for treatment of relapsed and refractory B-cell acute lymphoblastic anemia (B-ALL) with CTL019 (tisagenlecleucel-T), making it the first CAR construct to be submitted for regulatory approval (Kingwell, 2017). Moreover, Kite Pharma announced shortly thereafter (3/21/17) that they had completed their rolling BLA submission for treatment of non-Hodgkin lymphoma (NHL) using KTE-C19 (axicabtagene ciloleucel). If either CTL019 or KTE-C19 is, or both are, approved within the next year, it will mark a huge milestone in this exciting new field.

## DELIVERY OF ANTIBODIES TO NOVEL COMPARTMENTS

An area that has been of interest for many years, but has proven challenging, is the targeting of antibodies to compartments into which they do not normally go. These include, for examples, targeting antibodies to the gut via an oral route, to the brain by crossing the blood-brain barrier, or to the cytosolic intracellular compartment. All of these compartments present significant challenges, but in the past few years, significant strides have been made for all of them.

The most advanced tissue-targeted antibody-based product the bone-targeted enzyme replacement-Fc fusion, asfotase alpha (Strensiq®), which was approved by the US FDA for treatment of hypophosphatasia (Hofman et al., 2016). Asfotase alpha (TNSALP-Fc-deca-aspartate fusion protein) is targeted to bone with a deca-aspartate peptide fused to the C-terminus of the Fc (Millan et al., 2008).

The second area of antibody targeting that is represented by clinical candidates is based on the route of delivery to get the antibodies to the desired compartment. At least three orally-delivered, antibody-related proteins targeted to the intestinal tract are currently being evaluated in clinical trials. These include PRX-106 (Protalix®), an anti-TNF plant cell-expressed and delivered Fc fusion protein in phase II clinical trials (NCT02768974) for the treatment of ulcerative colitis (Ilan et al., 2017). The plant cells are thought to protect the Fc fusion while traversing through the stomach. Over the last decade, the mouse anti-CD3E mAb, OKT3, has been evaluated in clinical trials for oral delivery to the gut for treatment of nonalcoholic steatohepatitis (NASH; NCT01205087), with results suggestive of clinical activity (Lalazar et al., 2015). A second anti-CD3E mAb formulated for oral delivery is foralumab (NI-0401; NovImmune, Tiziana Life Sciences), a fully human mAb currently being prepared for phase II clinical trials for oral delivery for the treatment of NASH.

Getting antibodies to cross the blood-brain barrier has been a goal for well over two decades. IgG levels in the human brain are approximately 0.1% of the serum concentration of 9–10 mg/mL (Abbott et al., 2010). This differential is due to the blood-brain barrier (BBB) which effectively keeps antibodies out of the brain. Considering the wealth of potential targets for biologics in the central nervous system (CNS), there has been a great effort to find mechanisms to improve the ability to translocate biologics into the CNS. In recent years, significant progress has been made in getting antibodies to traverse the BBB. Yu et al. (2011) used a bivalent, bispecific antibody binding TFRC (transferrin receptor, CD71) with one arm and BACE1 (β-secretase-1) with the other arm, to demonstrate that low affinity antibodies to TFRC were more efficient at transcytosis than high affinity antibodies. While they only achieved about 12-fold higher accumulations of antibody in the brain over controls, they clearly demonstrated anti-BACE1 pharmacological activity of the antibody, proving that the antibody had accumulated within the brain (Yu et al. 2011). They also generated a bivalent, bispecific antibody targeting human and non-human primate (NHP) TFRC with one arm and human BACE1 with the other arm (Yu et al., 2014). The best variants, which were low-to-moderate affinity antibodies to TFRC, were accumulated 15-fold higher in the brain than control antibodies and they demonstrated *in vivo* pharmacological activity in NHPs (Yu et al., 2014).

Neiwoehner et al. (2014) compared the efficiency of transcytosis using a tetravalent, bispecific antibody with two arms each binding to TFRC and APP (amyloid-beta, Aβ) to a trivalent, bispecific antibody with only one arm binding TFRC. They found that monovalent binding to TFRC promoted efficient transcytosis whereas bivalent binding to TFRC resulted in shuttling the complex towards lysosomal degradation. They demonstrated a 55-fold improvement in target engagement over the control (Neiwoehner et al. 2014). In contrast to these studies in which monovalent targeting of the transcytotic receptor was optimal, the anti-TMEM30A (α(2,3)-sialoglycoprotein), llama single-domain antibody, FC5 (Abulrob et al., 2005), appeared to be transcytosed more efficiently as a dimer rather than a monomer (Farrington et al., 2014). Recently, FC5 was fused in an scFv format to the N-terminus of the HC of an anti-GRM (glutamate metabotropic receptor 1, mGluR1) antagonist IgG to shuttle it across the BBB (Webster et al., 2016), achieving pharmacological activity with a 10-fold enrichment of the antibody in the brain parenchyma (Webster et al., 2016). Thus, it still appears that there is much to be learned about optimizing antibodies for transcytotic delivery of proteins to the CNS.

William Pardridge and his colleagues have isolated an anti-human INSR (insulin receptor) antibody that can be transcytosed by INSR on endothelial cells lining the vasculature in the brain (Boado et al., 2007). They have used the anti-INSR antibody as a transcytotic carrier to move enzymes across the BBB for CNS enzyme replacement therapy (ERT) (Boado et al., 2012, 2014). These candidates are constructed by fusion of the enzymes to the C-terminus of the BBB-traversing anti-INSR IgG “HIRMAb” (Boado et al., 2012, 2014). AGT-181, which is a tetravalent (two antibody arms and two enzymes) fusion of an anti-INSR antibody and α-L-iduronidase (ALI) (Boado et al., 2012), is being evaluated in phase I clinical trials (NCT02371226) for the treatment of mucopolysaccharidosis I (MPS I; Hurler Syndrome). AGT-181 was recently demonstrated to be taken up by non-human primate brain at 1.2% of injected dose as compared to 0% injected dose of α-L-iduronidase alone (Boado and Pardridge, 2017), demonstrating the pharmacological relevance of the BBB-traversing bispecific antibody. AGT-182, comprised of a fusion of iduronate 2-sulfatase (IDS) to the C-termini of the anti-INSR HCs (Boado et al., 2014), is under phase I clinical testing (NCT02262338) for the treatment of mucopolysaccharidosis II (MPS II; Hunter Syndrome).

The final delivery-related technology that has gotten very interesting in recent years is the delivery of mAbs to the cytosol of cells via pinocytosis and endosomal escape (Marschall et al., 2014; Lönn et al., 2016; Stewart et al., 2016; Lim et al., 2017). Multiple approaches have been taken to get biologically active antibodies into the cytosol of cells, including the use of cell penetrating peptides (Marschall et al., 2014; Lönn et al., 2016; Lim et al., 2017). Just recently, a unique antibody has been generated for the delivery of an IgG to the cytosol of cells via endosomal escape (Choi et al., 2014). This antibody, which has a unique sequence in its light chain variable region, has been matured to increase the proportion of IgG that enters the cytoplasm (Kim et al., 2016). This, and other cell penetration technologies (Marschall et al., 2014; Lönn et al., 2016; Lim et al., 2017) bring hope that one day, antibodies will be used to target cytosolic antigens.

## NEW FORMS OF DELIVERY OF ANTIBODY GENES (DNA, RNA, AAVs, ONCOLYTIC VIRUSES)

Traditional forms of delivery for mAbs and Fc fusion proteins has been via either intravenous (IV) or subcutaneous (SC) administration of formulated proteins. Generally, high dose mAbs for oncology indications are limited to IV dosing, whereas low dose antibodies such as adalimumab, golimumab, and ustekinumab can easily be delivered in SC doses. Additionally, in recent years there has been increased interest in intratumoral dosing of antibodies and other biologics for certain types of cancer where the tumor is more accessible (Zeltsman et al., 2016). A novel approach for delivering mAbs and/or Fc fusion proteins is via delivery of the gene or genes that produce them, either as naked DNA, RNA, or by a viral-based vector. This is not an entirely new approach, since studies were done around the turn of the century showing that RNA (Giraud et al., 1999) and viral (Lewis et al., 2002) delivery of IgG genes could result in demonstration of *in vivo* IgG activity. Nevertheless, there was not much interest until the past few years, when it has become evident that vectored or nucleic acid delivery of IgG could potentially be a significant new approach to deliver antibodies for therapeutic use.

One of the more exciting forms of delivery is the intramuscular injection of adeno-associated viruses (AAVs) encoding antibodies, followed by years of consistently high expression of those antibodies in non-human primates (Fuchs et al., 2016; Greig et al., 2016). It is important to note that AAVs exist in the muscle cells as extrachromosomal elements and do not integrate, which increases the safety of their use for long term expression of antibodies or other proteins (Greig et al., 2016). This suggests that such an approach might be appropriate for delivery of anti-HIV antibodies to help patients either to become cured or, minimally, less reliant on highly active anti-retroviral therapy (HAART) (Schnepp and Johnson, 2014a; Fuchs and Desrosiers, 2016). There are several very promising, potent anti-HIV antibodies in clinical trials currently, some of which have been expressed *in vivo* using gene-based delivery of antibodies for potential therapeutic use (Schnepp and Johnson, 2014b; Yang and Wang, 2014; Fuchs et al., 2016; Fuchs and Desrosiers, 2016).

Similarly, but with a different twist, AAV-delivered antibodies to the nasal passages of mice have demonstrated excellent prophylaxis against flu virus (Limberis et al., 2013; Balazs et al., 2014; Adam et al., 2014). Since these AAVs enter epithelial cells that are sloughed off over several months, this provides a potentially safe route for delivery of prophylactic anti-flu antibodies that would cover the entire flu season. The potential significance of this approach is that there are several HA-binding and neutralizing antibodies available now that are nearly universal influenza virus inhibitors. These could potentially be used in clinical trials to determine whether or not this prophylactic, pan-influenza nasal delivery approach might be feasible.

Finally, the concept of using oncolytic viruses to deliver anti-tumor or checkpoint modulating antibodies to a tumor is very exciting. Oncolytic viruses have been engineered for years to deliver immune-modulating molecules such as CSF2 (GM-CSF) to the TME (Bommareddy et al., 2017), so it makes sense that they could be engineered to deliver TME modulating antibodies (Du et al., 2014). Several recent examples have demonstrated the potential for various types of oncolytic viruses expressing immune checkpoint inhibitors such as anti-PDCD1 (PD-1), anti-CD274 (PD-L1), and anti-CTLA4 (Du et al., 2014; Kleinpeter et al., 2016; Tanoue et al., 2017), as well as other anti-tumor antibodies (Adelfinger et al., 2015; Liikanen et al., 2016; Fajardo et al., 2017).

## SUMMARY

Over the past decade there has been a significant shift from discovery and development of basic antibodies, e.g., naked IgG1 isotype antibodies with no additional engineering other than perhaps humanization and affinity maturation, to more sophisticated forms of antibodies in all kinds of shapes and sizes. These newer forms include Fc-modified, glyco-engineered, bispecific, drug-conjugated, and cell surface expressed antibodies (i.e., CARs) as new weapons to fight difficult to treat diseases. We now see this dramatic shift in the types and numbers of modified antibodies now reaching clinical trial studies. This new phase of antibody drug discovery and development represents an exciting and bold new era that should see antibody-based therapeutics expanding their influence in many types of diseases. In the next few years we will likely see the first regulatory approvals of CAR-T based antibodies and immunocytokines, as well as approvals of additional new bispecific antibodies, new ADCs, Fc engineered antibodies, and glyco-engineered antibodies. Additionally, we should see new advances in targeting antibodies to the CNS and intracellular compartments, as well as nucleic acid or viral-vectored delivery. What an exciting time to be an antibody engineer!
